# Reassessment of IRES Element Activity in *Komagataella phaffii* Using a Standardized Bicistronic System: Cryptic Promoter Activity Mimics Cap-Independent Translation

**DOI:** 10.3390/ijms27188166

**Published:** 2026-09-14

**Authors:** Danil S. Kalinin, Ekaterina V. Schetinina, Alexey N. Fedorov, Igor E. Granovsky

**Affiliations:** 1 A.N. Bach Institute of Biochemistry, Research Center of Biotechnology of the Russian Academy of Sciences, Moscow 119071, Russia; 309163@gmail.com (D.S.K.); yekaterina.sh04@mail.ru (E.V.S.); a.fedorov@fbras.ru (A.N.F.); 2 Pushchino Scientific Center for Biological Research of the Russian Academy of Sciences, G.K. Skryabin Institute of Biochemistry and Physiology of Microorganisms of the Russian Academy of Sciences, Pushchino 142290, Moscow Region, Russia

**Keywords:** *Komagataella phaffii*, *Pichia pastoris*, internal ribosome entry site (IRES), cap-independent translation, bicistronic mRNA, cryptic promoter activity, polycistronic expression

## Abstract

Internal ribosome entry site (IRES) elements have been proposed for constructing polycistronic mRNAs in *Komagataella phaffii*, but their reported activity is inconsistent and has not been evaluated under standardized conditions. Here, a standardized bicistronic EGFP-IRES-mKate2 system was developed in which the cap-dependent first cistron (EGFP) reports promoter activity, and translation of the second (mKate2) should depend on the IRES. The RhPV, TEV, PVY, and *Saccharomyces cerevisiae* GPR1 elements were assessed under P_AOX1_ and P_THI11_, with EMCV and HCV as negative controls. EGFP tracked promoter induction and repression, whereas mKate2 remained at background for GPR1, EMCV, and HCV; for RhPV, TEV, and PVY it was low, promoter-independent, and persisted after promoter deletion. By RT-qPCR, the transcript was intact across both cistrons, except in the RhPV construct, where its 3′ region was depleted. The residual synthesis therefore reflected cryptic promoter activity below 1% of that of P_AOX1_, not IRES-dependent initiation. None of the elements directed detectable cap-independent translation above 0.4% of cap-dependent expression of the same ORF, limiting their use for polycistronic expression. The system, the first to place IRES activity in *K. phaffii* on a quantitative scale, distinguishes IRES-dependent translation from cryptic transcription and can facilitate the search for functional elements.

## 1. Introduction

In eukaryotes, most mRNAs are translated by a cap-dependent mechanism, in which the 5′-terminal cap (7-methylguanosine) is recognized by the translation initiation factor eIF4E, leading to recruitment of the small ribosomal subunit and scanning of the 5′ untranslated region (5′ UTR) in search of the AUG start codon [1]. In addition, an alternative, cap-independent pathway exists, namely internal translation initiation mediated by IRES (internal ribosome entry site) elements. These *cis*-acting RNA structures allow the ribosome to bind directly to an internal region of the mRNA, bypassing the requirement for the cap and, in some cases, for a subset of the canonical initiation factors [2]. IRES elements were first discovered in picornaviruses and were subsequently identified in a number of cellular mRNAs, including those of the yeast *Saccharomyces cerevisiae*, which indicates the conservation of this mechanism in eukaryotes [3,4,5].

The methylotrophic yeast *Komagataella phaffii* (*Pichia pastoris*) is widely used for metabolic engineering and synthetic biology owing to the ease of genetic manipulation and its capacity for post-translational modifications. However, the expression of heterologous metabolic pathways, which as a rule requires the co-expression of multiple genes, is technically demanding: conventional approaches employing several promoters or vectors are hampered by genetic instability due to homologous recombination and by the limited number of available selection markers [6,7,8]. In this context, the use of IRES elements to create polycistronic constructs, in which several genes are transcribed from a single promoter, appears to be an attractive solution.

To date, the functionality of IRES elements in *K. phaffii* has been investigated in several key studies. Liang et al. [5] were the first to report an IRES-dependent mechanism of translation initiation in *K. phaffii*, using a bicistronic construct under the control of the constitutive GAP promoter in which the first (RML lipase) and second (*lacZ*) cistrons were separated by the 5′ UTR of the *S. cerevisiae* GPR1 gene. They reported that *lacZ* expression depended on this insert and was not associated with cryptic promoter activity or splicing. Later, Xu et al. [9] reported the successful use of the encephalomyocarditis virus (EMCV) IRES in a bicistronic system under the methanol-inducible AOX1 promoter for the simultaneous expression of the *vgb* and *hph* genes, with IRES functionality inferred by the resistance of transformants to hygromycin B. The most extensive study was that of Huang et al. [6], in which a systematic screen of 34 IRES elements was carried out: to eliminate background transcription of full-length *lacZ*, the authors developed a system based on α-complementation of β-galactosidase, in which a bicistronic mRNA under the control of the AOX1 promoter encoded EGFP and the LacZ α-peptide, while the recipient strain constitutively expressed the ω-peptide. The screen identified seven IRES elements as functional: those of the TEV, PVY, RhPV, TRV, KSHV, and crTMV viruses, and the 5′ UTR of the *S. cerevisiae* YAP1 gene, whereas the activity of the EMCV and hepatitis C virus (HCV) IRES elements was not detected in this system [6].

The conflicting data on the EMCV IRES may be explained by differences in experimental design, both in the detection systems (full-length LacZ versus α-complementation) and promoters (GAP versus AOX1) and in the very principle of signal recording. In particular, the hygromycin selection method used by Xu et al. is indirect and highly sensitive: even a level of resistance-gene translation below the threshold of enzymatic detection may be sufficient for cell survival, which does not rule out a false-positive result, including one arising from cryptic promoter activity within the IRES sequence. The problem of artifacts is substantial in the field of IRES research: many cellular IRES elements identified initially were subsequently attributed to artifacts [1,10,11].

Thus, despite the accumulated data, there is no quantitative comparison of the known functional IRES elements under uniform, standardized conditions, supplemented by controls that would make it possible to reliably distinguish IRES-dependent translation from artifacts of cryptic transcription. Such a comparison is necessary both for resolving the existing contradictions and for assessing the applicability of IRES elements in the construction of polycistronic vectors.

Viral IRES elements are divided into four classes (1–4) that differ in their requirement for canonical initiation factors, degree of structuring, and mode of positioning the ribosome on the start codon; potyviral 5′-UTR elements and cellular IRESs fall outside this scheme and are considered separately [1,12] (Supplementary File S1, Table S1). The most factor-dependent is class 1, which requires almost the complete set of canonical eIFs and obligatory ITAFs; it has not been characterized in yeast and was not examined in the present work. For comparison, IRES elements differing in taxonomic affiliation, structure, and initiation mechanism were selected. The most compact are the IRES elements of two plant potyviruses: in tobacco etch virus (TEV), the IRES function is performed by a 143-nucleotide element whose activity critically depends on a pseudoknot and which requires the factor eIF4G rather than eIF4E [13,14]; the IRES of potato virus Y (PVY) is an AU-rich 5′ UTR (184 nt) that does not form stable secondary structures [15]. Both elements were reported to show high activity in the screen [6]. A special position is occupied by the dicistrovirus RhPV, whose intergenic (IGR) IRES (class 4), one of the most compact elements known (about 190 nt), initiates translation without protein factors by binding the ribosome directly, which in yeast is mediated by the ribosomal protein RPS25 [16,17]. As a negative control, the classical animal-virus IRES elements EMCV (class 2) and HCV (class 3) were chosen; these are exceptionally efficient in mammalian cells but, according to the available data, are inactive or show inconsistent activity in *K. phaffii* [6,9]. Finally, a cellular element was included in the set: the 5′ UTR of the *S. cerevisiae* GPR1 gene, whose IRES activity was reported in *K. phaffii* [5], but which was not part of the screen [6].

The aim of the present work was a rigorous reassessment of the activity of the TEV [14,18,19,20], PVY [21,22], RhPV (IGR) [16,23,24,25,26], EMCV [27,28], and HCV IRES elements [29,30,31] and the GPR1 5′ UTR [32] in a standardized bicistronic system of *K. phaffii*. To distinguish genuine IRES-dependent translation from artifacts of cryptic transcription, expression of the second cistron was assessed over time relative to the cap-dependent reporter of the first cistron, as well as in constructs with a deletion of the upstream promoter.

## 2. Results

### 2.1. Design and Construction of the Expression Cassettes and Selection of Recombinant K. phaffii Strains

In the first stage, bicistronic expression cassettes were constructed in which transcription of the marker genes is controlled by the methanol-inducible promoter P_AOX1_. Each cassette comprised, in the following order, the green fluorescent protein gene EGFP, the IRES element under study, and the red fluorescent protein gene mKate2.

The constructs were designed according to the following scheme. EGFP serves as a reporter of P_AOX1_ promoter activity; it is the first cistron, which is translated by a cap-dependent mechanism and reflects the overall level of transcription of the expression cassette. mKate2 serves as a reporter of IRES activity, since its translation is possible only if the IRES provides internal initiation at the start codon of the second cistron.

**Figure 1:**
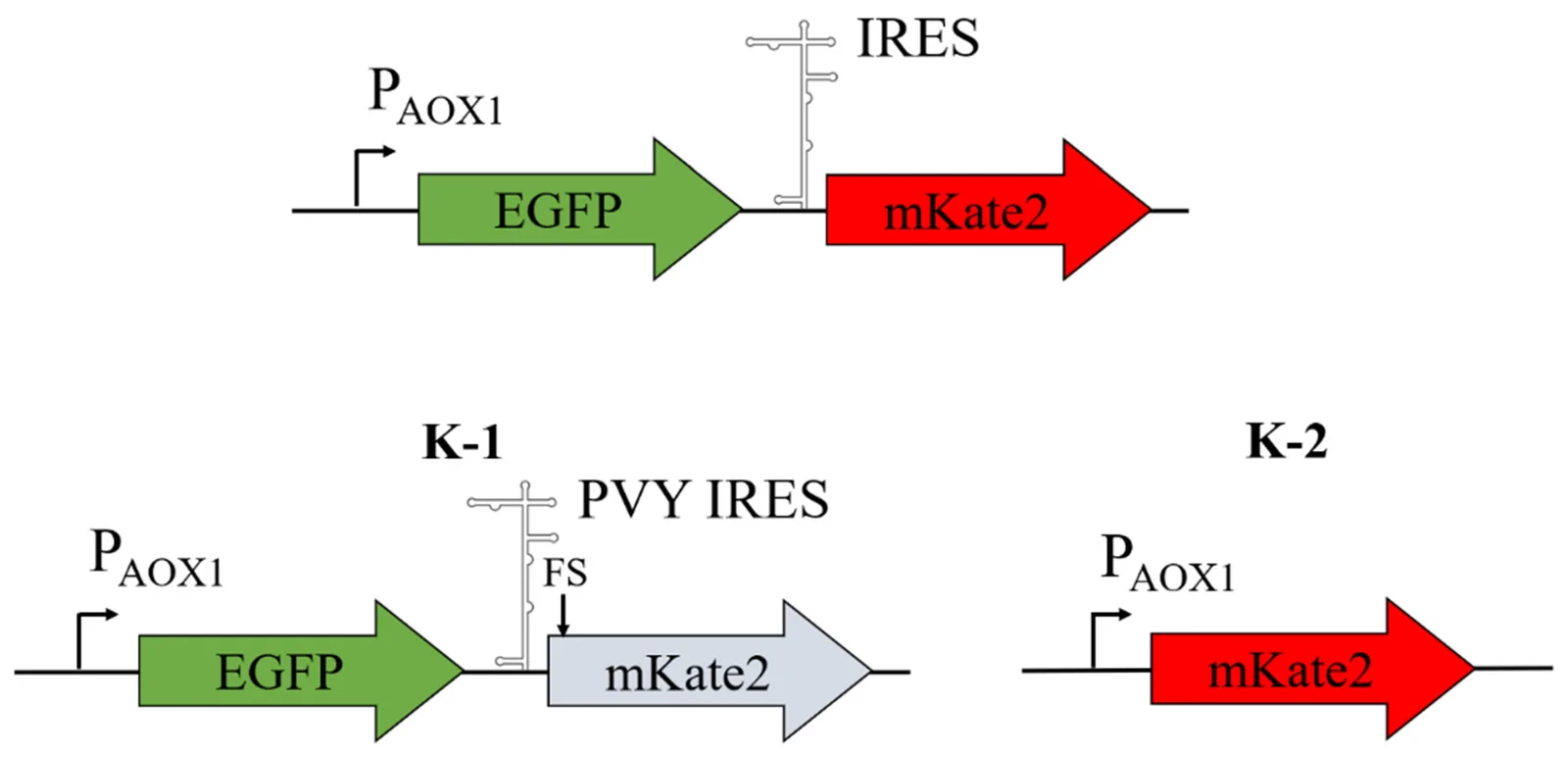
Schematic of the genetic constructs with expression cassettes under the control of the P_AOX1_ promoter. The upper part of the figure shows the bicistronic construct EGFP-IRES-mKate2, in which IRES is one of the IRES elements analyzed, namely those of the TEV, PVY, EMCV, HCV, and RhPV viruses and the *S. cerevisiae* GPR1 mRNA. The lower part of the figure shows the control expression cassettes under the control of the P_AOX1_ promoter: K1, a bicistronic cassette isogenic to EGFP-IRES-mKate2 but carrying a − 1 frameshift mutation that inactivates the mKate2 ORF, so that only EGFP is functionally expressed (the single-nucleotide deletion is indicated by a vertical arrowhead); and K2, a monocistronic cassette with the mKate2 ORF.

In this work, the IRES elements of the TEV, PVY, and RhPV viruses, as well as the IRES element of the *S. cerevisiae* GPR1 mRNA, were investigated. As a control for cap-dependent expression of the first cistron, construct K1 (p9KAox-G) was used; it is isogenic to the bicistronic cassette EGFP-IRES-mKate2 but carries a − 1 frameshift mutation in the mKate2 ORF, as a result of which only EGFP is functionally expressed. The control for cap-dependent expression of mKate2 was the monocistronic construct K2 (p9KAox-K), in which the mKate2 ORF was under the direct control of P_AOX1_ (Figure 1). In addition, as a negative control, constructs with the IRES elements of the mammalian viruses EMCV and HCV were used; these have been shown to be unable to support internal cap-independent translation initiation in yeast [6].

The resulting constructs were integrated into the *K. phaffii* chromosome, and in the selected clones we verified that the cassettes had integrated correctly and at a defined copy number. The genomic region spanning the integrated cassette was amplified with primers annealing to the AOX1 promoter and terminator (Section 4), which co-amplify the integrated cassette together with the parental AOX1 locus; all six PAOX1 constructs yielded the expected products (Figure S1 in Supplementary File S2). Nanopore sequencing of these fragments showed that the reads covered the entire length of each cassette, including the IRES region, at high mean depth (360×–1050×; at least 99% of the length at ≥10× coverage) (Figure S2; Table S4 in Supplementary File S2). The consensus sequences matched the designed cassettes (Supplementary File S2), confirming full-length, mutation-free integration; the only discrepancy, a single position within a homopolymeric A-tract of the GPR1 IRES, reflects the known difficulty of nanopore sequencing in resolving homopolymer length and was confirmed by Sanger sequencing of the plasmid.

In parallel, the copy number of the integrated cassette was determined by qPCR using the standard-curve method (Supplementary File S3, Table S2). In all six clones, the mKate2/GAPDH ratio was close to 1 (Supplementary File S3, Table S3), indicating a single integrated copy of the cassette, consistent with single-crossover integration at the *HIS4* locus. The first cistron gave a concordant result: the EGFP/GAPDH ratio likewise indicated a single copy, confirming that both cistrons of the cassette had co-integrated intact; its somewhat higher absolute value reflects the marginally higher amplification efficiency of the EGFP assay (Section 4).

### 2.2. Analysis of IRES Activity in Bicistronic mRNAs Synthesized from the AOX1 Promoter

Expression of the cassettes was induced by the addition of methanol as described in Section 4. Samples for analysis were taken at 0, 24, 48, 72, and 120 h. The fluorescence signal was detected using a flow cytometer. For each strain, the mean fluorescence value was calculated after subtraction of the background fluorescence signal of GS115 cells induced with methanol (Section 4; Supplementary File S4).

**Figure 2:**
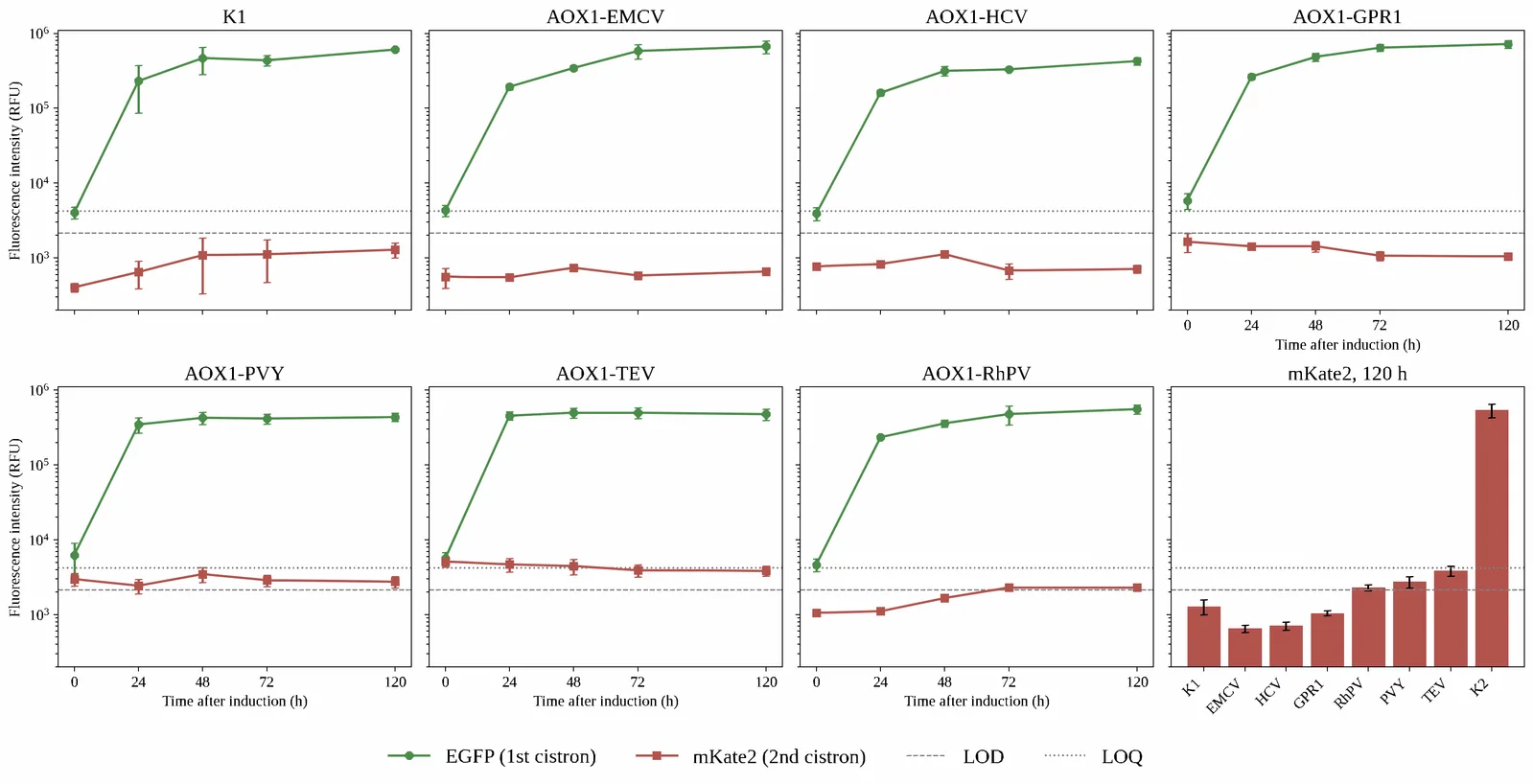
Time course of EGFP and mKate2 expression from mono- and bicistronic expression cassettes under the control of the P_AOX1_ promoter in *K. phaffii*. Each of the first seven panels shows the mean fluorescence intensity (relative fluorescence units, RFU; logarithmic scale) of the EGFP (green) and mKate2 (red) reporters at 0, 24, 48, 72, and 120 h of induction for one construct: K1, the EGFP-expression control; AOX1-EMCV, AOX1-HCV, AOX1-GPR1, AOX1-PVY, AOX1-TEV and AOX1-RhPV, bicistronic EGFP–IRES–mKate2 cassettes carrying the EMCV, HCV, GPR1, PVY, TEV and RhPV IRES elements, respectively. The lower-right panel (“mKate2, 120 h”) summarizes the mKate2 signal at 120 h for the same constructs together with the monocistronic mKate2 control K2, included as a positive reference (Supplementary File S4, Table S6). In all panels, the dashed and dotted lines mark the limit of detection (LOD, 2163 RFU) and the limit of quantification (LOQ, 4207 RFU) of the mKate2 signal, respectively (Section 4; Supplementary File S4, Table S5). Values are means ± SD (*n* = 3); host-strain (GS115) autofluorescence was subtracted from all values.

First, we determined the basic operating parameters of the proposed experimental model. To this end, methanol-inducible expression of the reporter genes was analyzed in the control construct K1 (plasmid p9KAox-G), in which the mKate2 ORF is inactivated by a − 1 frameshift mutation and only EGFP is functionally expressed (Section 4). Figure 2 shows the flow cytometry data for the recombinant *K. phaffii* strains in the green and red channels over the 120 h time course. Analysis of the fluorescence of construct K1 showed that, as expected, upon induction with methanol, the green fluorescence signal increased by more than two orders of magnitude, whereas the fluorescence signal in the red region of the spectrum remained at only ~0.2% of the green signal, reflecting green-to-red spectral bleed-through. This residual signal of strain K1 was taken as the blank for estimating the limit of detection (LOD) and the limit of quantification (LOQ) of the mKate2 signal (Section 4; Supplementary File S4, Table S5).

We next assessed the sensitivity of the chosen approach for measuring the fluorescence signal in the red region of the spectrum. For this purpose, we analyzed expression of the control construct K2 (plasmid p9KAox-K), in which the mKate2 ORF is placed within a monocistronic cassette under the direct control of P_AOX1_. As in the case of EGFP, induction with methanol increased the red fluorescence signal by more than three orders of magnitude. Relative to this positive reference, the LOD and LOQ correspond to 0.40% and 0.78%, respectively, of the mKate2 signal obtained for construct K2 (Supplementary File S4, Table S5); that is, the system detects authentic mKate2 over a dynamic range of about 250-fold above the LOD.

We then analyzed the expression of EGFP and mKate2 in *K. phaffii* cells carrying in the chromosome, bicistronic cassettes, in which the mKate2 ORF is preceded by the IRES of the EMCV or HCV viruses (plasmids p9KAox-GEK and p9KAox-GHK, respectively). The IRES elements of these viruses have been shown to be inactive in *K. phaffii*. For the EMCV and HCV constructs, classical methanol-dependent EGFP expression was likewise observed. However, the level of the red fluorescence signal, corresponding to mKate2 expression, did not exceed the LOD at any time point (Figure 2; Supplementary File S4). These results are consistent with the findings of Huang et al. [6] that these IRES elements are not functional in *K. phaffii*. For the construct containing the GPR1 IRES, mKate2 fluorescence likewise did not exceed the LOD at any time point and amounted to only about 0.15% of EGFP fluorescence (Figure 2; Supplementary File S4, Table S6).

For the remaining constructs, containing the RhPV, PVY, and TEV IRES elements, the mKate2 signal at 120 h exceeded the LOD, amounting to 0.41, 0.63, and 0.81% of the EGFP signal, respectively (Figure 2; Supplementary File S4, Table S6). That is, mKate2 was synthesized only in trace amounts; at 120 h the signal did not reach the LOQ for any of the constructs, and only for TEV did it approach that level, exceeding the LOQ at the early time points (0–48 h) and declining below it thereafter. On detailed analysis of the expression dynamics, we found that the accumulation of mKate2 over time did not follow that of EGFP. Upon the addition of methanol, the green EGFP signal increased 70- to 120-fold, as expected, whereas over the same period the mKate2 signal increased only ~2-fold (RhPV) or did not increase at all, remaining essentially constant (PVY) or even decreasing slightly (TEV) (Figure 2). This discrepancy cast doubt on the interpretation of the observed mKate2 signal as a result of IRES-dependent translation.

**Figure 3:**
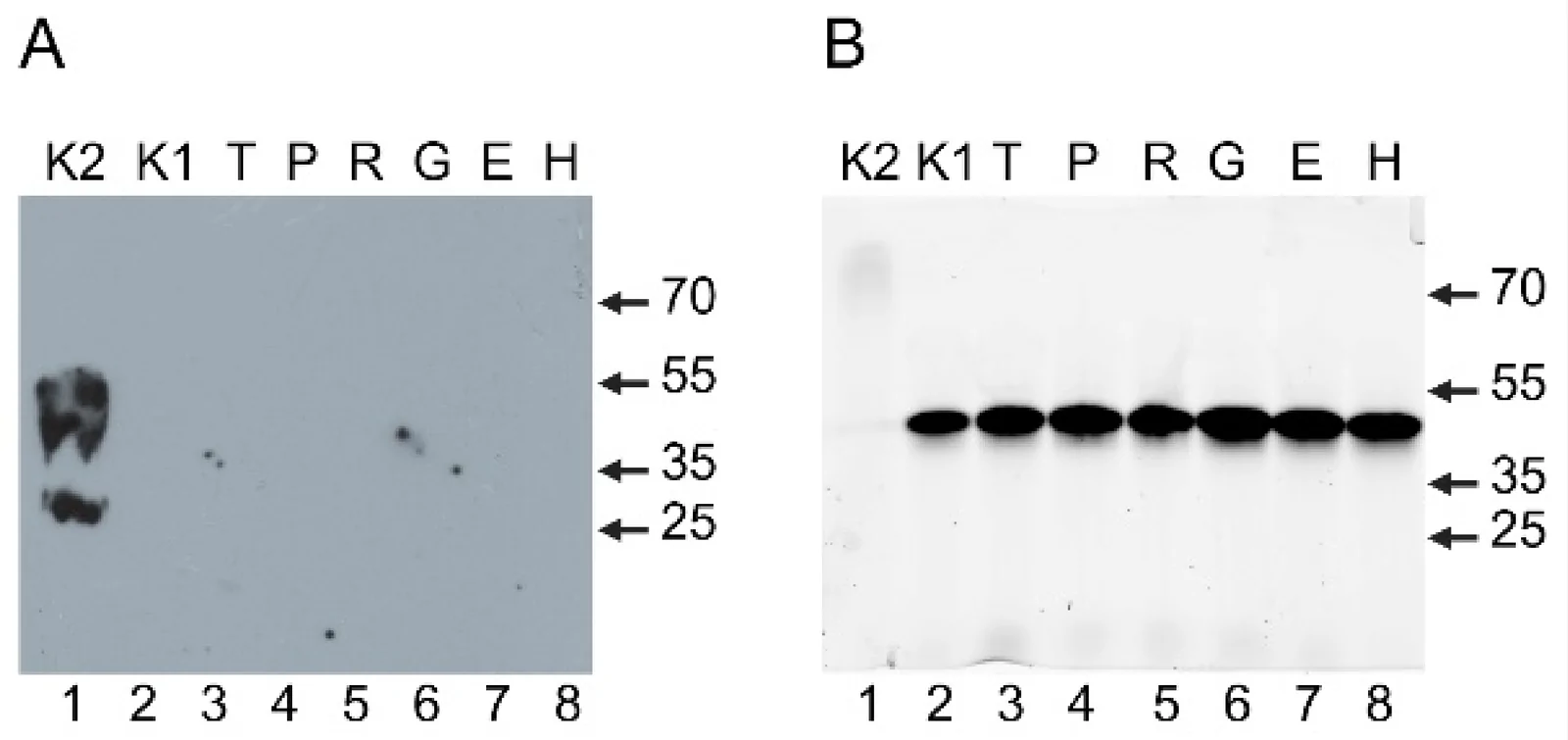
Production of the mKate2 protein and expression of EGFP in *K. phaffii* cells carrying mono- and bicistronic expression cassettes under the control of the P_AOX1_ promoter. (**A**) Immunoblot analysis of lysates of induced cells with anti-mKate2 antibodies (Section 4). (**B**) In-gel fluorescence of EGFP after separation of lysates of the same cells by non-denaturing PAGE, recorded on a Typhoon FLA 9500 imager (Section 4). Lanes (for **A** and **B**): K2, monocistronic mKate2; K1, EGFP-expression control (with a frameshift in the mKate2 ORF); T, P, R, G, E, H, bicistronic constructs carrying the TEV, PVY, RhPV, GPR1, EMCV, and HCV IRES elements, respectively. Arrows indicate the positions of molecular weight markers in kDa. In panel B, the marker positions are shown for orientation only, since electrophoretic mobility under non-denaturing conditions does not reflect molecular mass.

To determine whether the presence or absence of the red fluorescence signal corresponded to the accumulation of mKate2 in the cell, we analyzed its content by immunoblotting with antibodies against this protein (Section 4). As is evident from the results of this analysis (Figure 3A), mKate2 was detected only in cells carrying the monocistronic construct K2, where it migrated as a band of the expected electrophoretic mobility; the additional band of higher apparent mass corresponds to an incompletely denatured form, characteristic of fluorescent proteins. Transfer quality was confirmed by Ponceau S staining of the membrane (Supplementary File S2, Figure S3A). In all the other samples, mKate2 was absent and was not detected—either as the full-length protein or as shorter antibody-reactive polypeptides—even upon longer exposure (Supplementary File S2, Figure S3B). In the same samples, we also examined the presence of EGFP by in-gel detection of the green fluorescence signal (Section 4). As shown in Figure 3B, EGFP was reliably detected in all cells except those carrying the monocistronic construct K2. Its level was similar in all EGFP-expressing strains, the small differences in in-gel fluorescence being consistent with the amount of total cell protein loaded onto the gel (Supplementary File S2, Figure S4). It can therefore be concluded that the very low red fluorescence signal observed in *K. phaffii* cells carrying the bicistronic constructs reflects a correspondingly low amount of mKate2 protein in the cells rather than impaired maturation or truncation of the protein.

### 2.3. Analysis of the Intramolecular Integrity of the Bicistronic mRNA

The immunoblot analysis described above showed that the low mKate2 fluorescence recorded by flow cytometry in cells carrying the bicistronic constructs corresponds to a genuinely low amount of the protein. In principle, this could arise not only from inefficient translation of the second cistron, but also from the transcript itself: if the bicistronic mRNA were shortened or processed within the cassette, the mKate2-coding region would be lost from it, and the second cistron would be absent from the template rather than merely untranslated. Since both cistrons are transcribed from a single promoter as a single mRNA, the two markers can be used to probe the same molecule: quantification of the 5′ (EGFP) and the 3′ (mKate2) regions in the same RNA preparation reports their relative abundance within the transcript. Total RNA was therefore isolated 48 h after induction, the same time point as for the immunoblot analysis, and the two regions were quantified by reverse-transcription qPCR with marker-specific primer pairs, the reverse primer of each pair annealing within the 3′-terminal (stop-codon) region of the corresponding ORF (Section 4).

Because the two regions are measured with different primer pairs, the absolute value of ΔCp = Cp (mKate2)—Cp (EGFP) is not informative in itself; what is informative is the comparison between the constructs. For five of the six constructs, those carrying the TEV, PVY, EMCV, HCV, and GPR1 elements, ΔCp fell within a narrow range of 0.7–1.7 cycles (Table 1), which thus defines the baseline offset of the assay. This difference is negligible, given the somewhat higher amplification efficiency of the EGFP assay compared with that of mKate2 (Section 4; Supplementary File S3, Table S2). Consequently, the 5′ and the 3′ regions of the bicistronic mRNA were present in comparable amounts, indicating that in these strains the transcript remains intact across both cistrons and that the absence of mKate2 cannot be attributed to the loss of the second cistron from the mRNA. It should be noted that the assay compares the abundance of the 5′ and the 3′ regions and would not reveal internal processing events that retain both markers. However, no canonical *S. cerevisiae*-type 5′ splice-site donor or branch-point motifs, both of which are required for intron recognition and splicing in yeast, were found in any of the six IRES sequences examined (Section 4), so cryptic splicing within the insert is unlikely.

The construct carrying the RhPV element behaved differently: ΔCp reached 5.1 ± 0.2 cycles. Given that the mean ΔCp for the other five constructs was ≈1 and that the Cp value for the EGFP marker did not differ among all six strains, the ΔCp of the RhPV construct can be corrected for this offset to approximately four cycles, which corresponds to an about 16-fold lower abundance of the 3′ (mKate2) region relative to the 5′ (EGFP) region of the same transcript. The observed effect therefore reflects a specific depletion of the 3′ portion of the bicistronic mRNA. In control reactions performed without reverse transcriptase, amplification was detected 11.2–16.6 cycles later than in the corresponding complete reactions (Table 1), confirming that the signals, including the low mKate2 signal of the RhPV strain, originated from RNA and not from residual DNA.

To establish whether the loss of the 3′ region could result from processing of the transcript in the nucleus, the sequence of the RhPV insert was screened in silico for cryptic *S. cerevisiae*-type RNA-processing signals: the 5′ splice-site and branch-point consensus motifs for splicing, and the UA-rich efficiency element together with the downstream A-rich positioning element for polyadenylation and transcription termination (Section 4; Supplementary File S1, Table S7).

No canonical 5′ splice-site donor was present in the RhPV insert, and the branch-point motif was matched only with a single mismatch, so a splicing origin of the effect is not supported. The insert did contain three UA-rich efficiency-element-like motifs, namely UAUAUA at positions 227–232, 318–323, and 523–528 of the 537-nt insert, together with a single A-rich positioning-element-like motif (AAUAAA at positions 200–205). These motifs, however, do not constitute a canonical tripartite yeast polyadenylation and termination signal: none of the UA-rich motifs is followed by an A-rich positioning element within the ~10–40-nt window characteristic of such signals, the search having been extended beyond the insert into the mKate2 ORF, and the only A-rich motif lies upstream of all three UA-rich motifs (Supplementary File S1, Table S7).

The mKate2 ORF itself can be excluded as the source of the effect: it is identical, together with its 3′ flanking region, in all six bicistronic cassettes, yet depletion of the 3′ region was observed for RhPV alone; in addition, the same ORF is efficiently expressed in the monocistronic construct K2, in which its 3′ end is the same as in the bicistronic cassettes. The loss of the 3′ region is therefore determined by the RhPV insert. Taken together, these results show that in five of the six constructs the bicistronic mRNA is intact across both cistrons, whereas the construct carrying the RhPV element is a special case, in which the 3′ portion of the transcript is specifically depleted; no canonical cryptic splicing or polyadenylation signal accounting for this was identified in the RhPV sequence, and establishing the exact cause of the loss of integrity requires further study.

### 2.4. Analysis of IRES Activity in Promoterless Constructs

Since the bicistronic transcript proved to be intact across both cistrons in five of the six constructs (Section 2.3), the low level of mKate2 cannot be attributed to the loss of the second cistron from the template. The absence of a correlation between the accumulation dynamics of EGFP and mKate2 therefore allowed us to put forward an alternative hypothesis: mKate2 synthesis may result not from IRES-dependent translation but from the presence of intrinsic promoter activity in the DNA sequences encoding the viral IRES elements.

**Figure 4:**
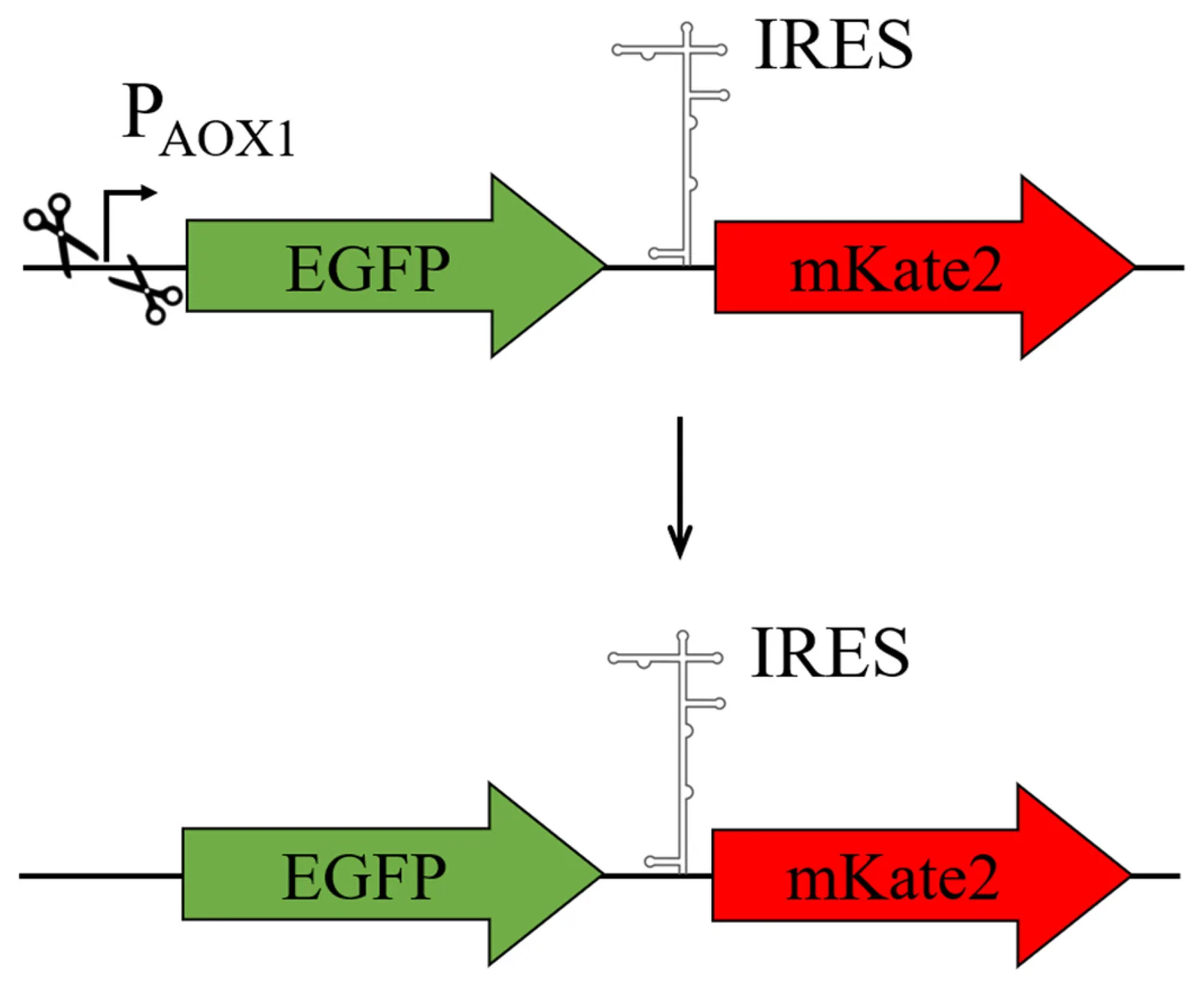
Scheme for the generation of the genetic constructs with expression cassettes from which the PAOX1 promoter has been deleted. Plasmids pHik-GTK, pHik-GPK, and pHik-GRK were derived from p9KAox-GTK, p9KAox-GPK, and p9KAox-GRK, respectively, by deletion of the PAOX1 promoter; in all other respects, the cassettes are isogenic to the parental ones.

**Figure 5:**
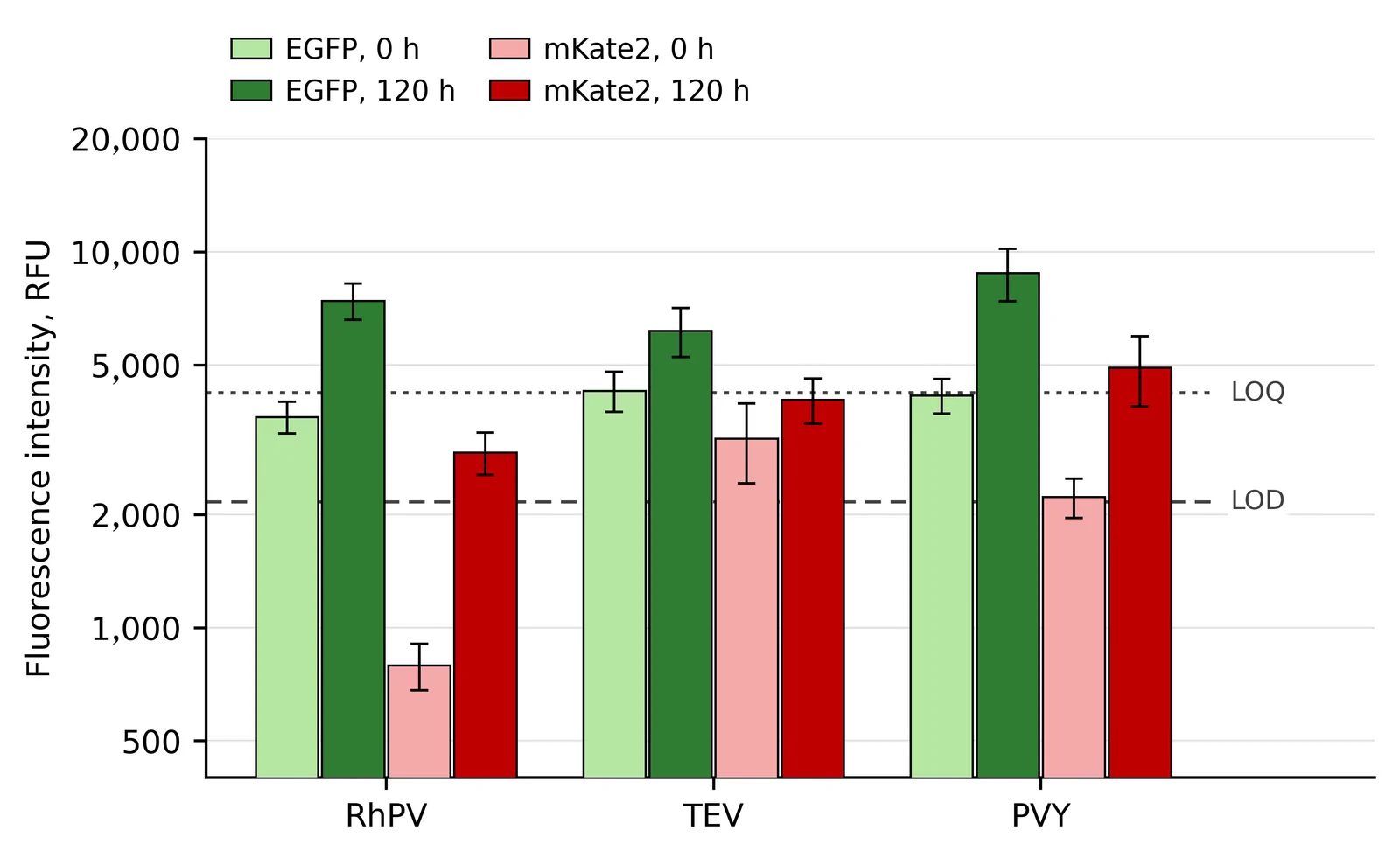
Analysis of EGFP and mKate2 ORF expression in *K. phaffii* within bicistronic cassettes in constructs from which the PAOX1 promoter has been deleted. Flow cytometry data are shown for the EGFP and mKate2 channels of recombinant *K. phaffii* cells transformed with the plasmids: RhPV, pHik-GRK; TEV, pHik-GTK; and PVY, pHik-GPK. The histogram shows fluorescence intensity on a logarithmic scale. RFU, relative fluorescence units. Green bars correspond to the EGFP signal and red bars to the mKate2 signal: light green and light red, 0 h; dark green and dark red, 120 h after transfer to methanol-containing medium. The dashed and dotted lines mark the limit of detection (LOD, 2163 RFU) and the limit of quantification (LOQ, 4207 RFU) of the mKate2 signal, respectively (Section 4; Supplementary File S4, Table S5). Values are means ± SD (*n* = 3); host-strain (GS115) autofluorescence was subtracted from all values.

To test this hypothesis, the external promoter P_AOX1_ was deleted from the bicistronic cassettes with TEV, PVY, and RhPV (Section 4; Figure 4). Thus, if mKate2 production were supported solely by IRES-dependent translation, it should cease in the absence of the promoter. The result of the expression analysis of these constructs was unequivocal: after deletion of the promoter, the red fluorescent protein continued to be produced in all three cases (Figure 5). While the EGFP signal fell to 1.3–2.0% of that in the corresponding P_AOX1_-driven strains, the mKate2 signal was not reduced (Supplementary File S4, sheet ∆AOX1). In all three promoterless constructs, the mKate2 signal at 120 h exceeded the LOD, and in the case of PVY it exceeded the LOQ (Supplementary File S4, Table S8). These thresholds are conservative for such strains, since they were derived from the blank measured at maximal EGFP expression (Supplementary File S4, Table S5), whereas the green-channel signal of the promoterless strains, and hence the spectral bleed-through into the mKate2 channel, is 50- to 80-fold lower. This suggests that the IRES sequences or the upstream sequences possess cryptic promoter activity in *K. phaffii*.

For quantitative assessment of the cryptic activity detected, the mKate2 signal was compared not with the EGFP signal but, as in Section 2.2, with the mKate2 signal in the monocistronic cap-dependent construct p9KAox-K, since EGFP and mKate2 differ substantially in their photophysical and maturation characteristics and their signals within a single cell are therefore not directly comparable. Normalization to the mKate2 signal in the p9KAox-K construct (data given in Supplementary File S4, sheet RELSC) showed that the cryptic promoter activity is very low and amounts to less than 1% of the activity of the PAOX1 promoter (Table 2).

### 2.5. Analysis of the Activity of the PVY and TEV IRES Elements Under Promoter Repression

**Figure 6:**
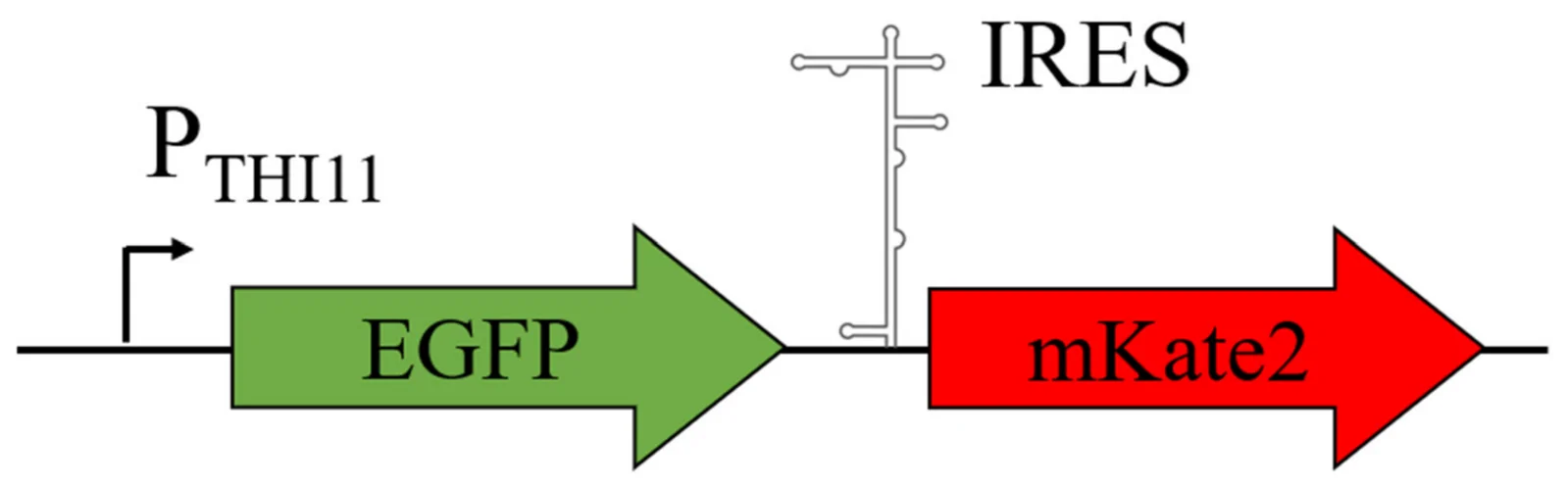
Schematic of the genetic constructs with expression cassettes under the control of the PTHI11 promoter. The bicistronic construct EGFP-IRES-mKate2 is shown, in which IRES is one of the IRES elements analyzed, that of the TEV (plasmid p9KTh11-GTK) or PVY (plasmid p9KTh11-GPK) virus.

To test the activity of the IRES elements independently of PAOX1 and under conditions in which transcription of the cassette is progressively switched off rather than induced, an additional experiment was performed. For this experiment, the TEV and PVY IRES elements were chosen as those that had shown the highest mKate2 signal under the control of PAOX1. The corresponding EGFP-IRES-mKate2 cassettes were placed under the control of the PTHI11 promoter (Figure 6). The activity of PTHI11 is regulated by thiamine: derepression of the promoter occurs as thiamine is depleted in the culture medium [33,34].

**Figure 7:**
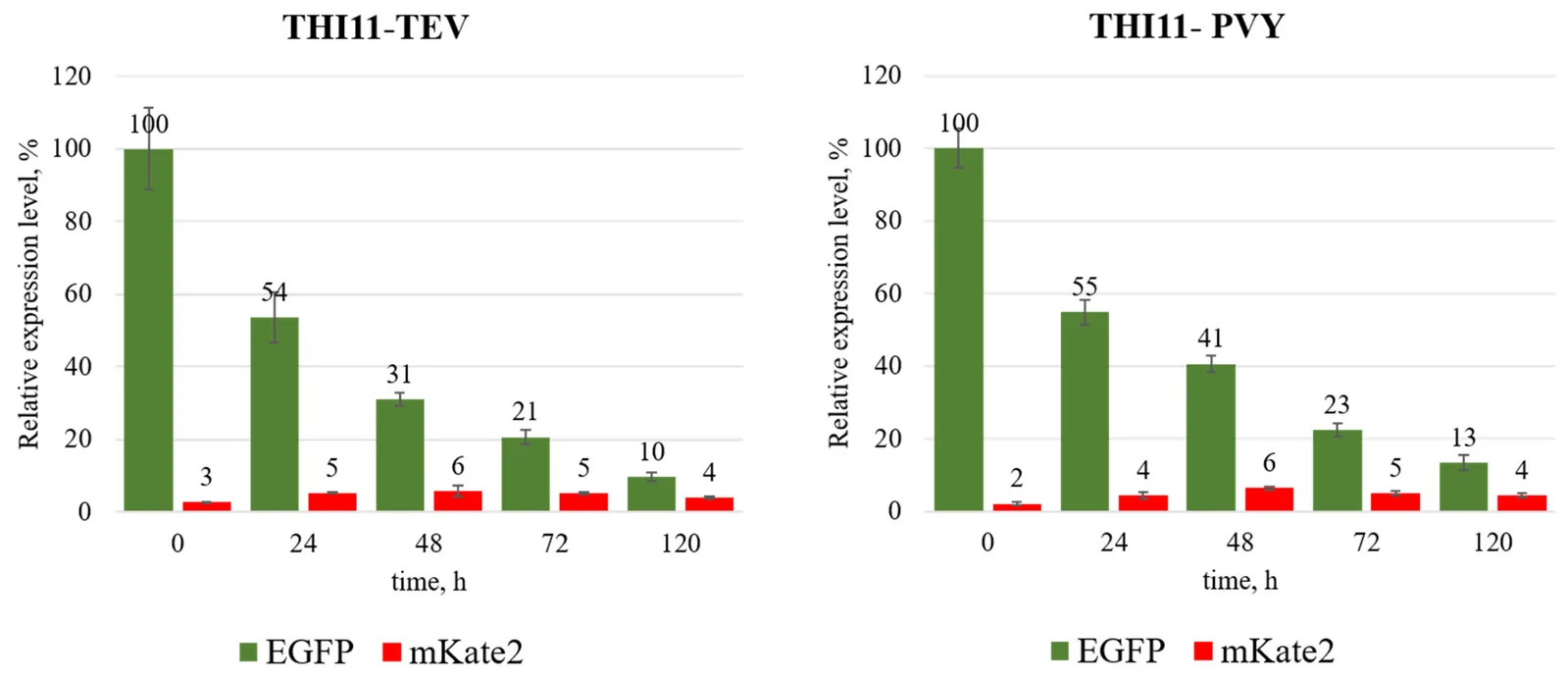
Dynamics of EGFP and mKate2 ORF expression in *K. phaffii* within bicistronic cassettes in constructs under the control of the PTHI11 promoter. Flow cytometry data are shown for the EGFP and mKate2 channels of recombinant *K. phaffii* cells transformed with the plasmids: TEV, p9KTh11-GTK; and PVY, p9KTh11-GPK. The histogram shows fluorescence intensity in relative fluorescence units (RFU). Green bars correspond to the EGFP signal and red bars to the mKate2 signal. Values are means ± SD (*n* = 3); host-strain (GS115) autofluorescence was subtracted from all values.

*K. phaffii* cells carrying the expression cassettes mentioned above were grown in thiamine-free medium, as described in Section 4, to induce the PTHI11 promoter. The cells were then transferred to a rich, thiamine-containing medium to repress its activity, and changes in the green and red fluorescence signals were monitored over time (Figure 7). As expected, after transfer to the thiamine-containing medium, a gradual decrease in the green EGFP fluorescence signal by ~10-fold was observed over 120 h, reflecting the repression of the transcriptional activity of the PTHI11 promoter and a decrease in the EGFP content of the cells. The mKate2 signal did not follow this decline: it increased about twofold over the first 24–48 h after repression of the PTHI11 promoter and thereafter declined only slightly, remaining above its initial level and above the LOQ at all time points from 24 h onwards (Supplementary File S4, Table S8). In the histogram (Figure 7), we used a linear scale of relative fluorescence units to demonstrate clearly how much weaker the expression of the mKate2 ORF is relative to that of the EGFP ORF driven by the same promoter, the strength of PTHI11 in our experiments for the isogenic cassettes amounting to ~30% of that of PAOX1 (Supplementary File S4, sheets AOX1 and THI11).

## 3. Discussion

Both the search for cellular IRES elements and the characterization of heterologous viral IRES elements are challenging tasks, the solution of which does not infrequently lead to artifacts. This is due to the need for a comprehensive study of the translation of IRES-containing mRNAs, directed primarily at excluding promoter activity of the putative IRES element and other upstream sequences, as well as possible splicing of transcripts in the nucleus to form alternative mRNAs. In this connection, it is not surprising that the results of some independent studies are in poor agreement with one another.

In our case, the results presented are consistent with the data of Huang et al. [6] with respect to the absence of EMCV and HCV activity in *K. phaffii* cells. The GPR1 IRES should be assigned to the same group: in our system, the mKate2 signal for it did not exceed the limit of detection (about 0.15% of the EGFP signal); that is, the activity previously reported by Liang et al. [5] was not reproduced. Thus, our results diverge from the reports on the functionality of EMCV [9] and GPR1 [5]. It should be noted that Liang et al. tested a promoterless construct for GPR1; however, the design of their control differed from ours: the authors used a monocistronic vector containing only GPR1 + *lacZ* without the first cistron. Such a control excludes autonomous promoter activity of the GPR1 sequence itself but does not allow the detection of cryptic transcription arising in the context of the complete bicistronic cassette. The deletion of the external promoter from the complete EGFP-IRES-mKate2 cassette that we applied is a more stringent control: it was precisely this deletion that revealed residual mKate2 synthesis in the constructs with the TEV, PVY, and RhPV IRES elements, indicating cryptic promoter activity of the upstream sequences. Taken together with the absence of a detectable GPR1 signal in our system, this suggests that the GPR1 activity reported by Liang et al. may also have been of artifactual origin.

Another source of the discrepancy between our results and the data of Liang et al. [5] and Huang et al. [6] is the method of detecting expression of the ORF under the control of the putative IRES element. In those studies, it was recorded through β-galactosidase activity, the signal of which accumulated throughout the incubation period and did not reflect changes in the content of the produced protein over time. Terenin et al. [1] note that recording at a single time point after transfection or induction, without analysis of the kinetics of reporter protein accumulation, may mask, in particular, differences in mRNA stability and thereby lead to artifacts in the assessment of IRES activity [1]. This may have been the cause of the discrepancy between our data and the results of Huang et al. [6], in which IRES activity was assessed through expression of the LacZ α-peptide followed by complementation of the β-galactosidase ω-fragment, with expression of *lacZ*α under the control of the constitutive promoter PGAP and enzyme activity determined either qualitatively (from accumulation of the X-Gal cleavage product over three days of colony growth) or quantitatively at a single point once the culture reached a certain optical density. The system we propose, in contrast, makes it possible to monitor the synthesis and accumulation of mKate2 over time and, importantly, relative to the accumulation dynamics of the reference protein EGFP, whose translation proceeds by a cap-dependent mechanism.

A further possible source of the low mKate2 level had to be excluded at the level of the transcript itself: if the bicistronic mRNA were shortened or processed within the cassette, the second cistron would be absent from the template rather than merely untranslated. Quantification of the 5′ (EGFP) and the 3′ (mKate2) regions of the transcript in the same RNA preparation showed that in five of the six constructs the two regions are present in comparable amounts (Section 2.3, Table 1); the transcript therefore remains intact across both cistrons, and the low level of mKate2 cannot be attributed to the loss of the second cistron from the mRNA. This conclusion is supported at the level of the sequence as well, since no canonical *S. cerevisiae*-type 5′ splice-site donor or branch-point motifs were found in any of the inserts examined. Splicing of the primary transcript is the mechanism that was shown to underlie the apparent activity of a number of cellular IRES elements [10,11], and it is also the artifact that Liang et al. [5] specifically excluded for the GPR1 5′ UTR; on this particular point, our data agree with their conclusion.

The construct carrying the RhPV element proved to be the exception: the 3′ region of its transcript was approximately 16-fold less abundant than the 5′ region (Section 2.3, Table 1). A selective loss of the 3′ portion of a transcript whose 5′ portion is retained points to processing of the transcript rather than to its degradation, but the signal responsible for it was not identified. It should be noted that the insert used here is longer than the IGR IRES element proper: the 537-nt fragment corresponds to the complete intergenic region of the RhPV genome (GenBank NC_001874) and thus contains the 220-nt sequence employed in the screen of Huang et al. [6] together with the remainder of that region; even within this extended fragment no canonical arrangement of a UA-rich efficiency element and a downstream A-rich positioning element was found (Section 2.3; Supplementary File S1, Table S7). For the same reason, our data do not bear directly on the activity reported for the shorter RhPV element: a processing signal located within the additional upstream sequence would not have been present in that construct. The search, however, relied on *S. cerevisiae* consensus motifs, whereas the signals of 3′-end processing in *K. phaffii* have not been characterized in comparable detail, so that a species-specific or non-canonical signal would have escaped detection. As a consequence, for the RhPV construct, our analysis cannot separate two contributions to the low level of mKate2—an inability of the element to direct internal initiation and a reduced amount of the template carrying the second cistron—and our conclusion for this element is therefore more cautious than for TEV and PVY. The reduced amount of template does, however, allow the negative result for this element to be given a quantitative bound. Where the transcript is intact, an element directing internal initiation is detectable at 0.40%, and quantifiable at 0.78%, of the cap-dependent expression of the same ORF (Section 2.2; Supplementary File S4, Table S5). Where the template carrying the second cistron is approximately 16-fold less abundant, these same thresholds correspond to an approximately 16-fold higher efficiency of the element itself, that is, to about 6% and about 13%, respectively. In this strain, no IRES-dependent component was distinguished within the mKate2 signal: it did not follow induction of P*AOX1* and was not reduced upon deletion of the promoter (Section 2.2 and Section 2.4). If the RhPV IGR IRES directs internal initiation in *K. phaffii* at all, its efficiency is therefore below approximately 6% of the cap-dependent expression of the same ORF—a bound sixteen-fold less stringent than the one obtained for the elements whose transcripts remained intact. Assessing the potential of the RhPV IGR IRES in *K. phaffii* will require establishing the exact mechanism by which the integrity of the mRNA is compromised in its presence.

According to the results of Huang et al. [6], activity was reported, in particular, for the RhPV, TEV, and PVY IRES elements, and it was highest for TEV and PVY. The TEV and PVY elements examined here correspond to the sequences used in that screen, so that for these two elements the comparison is direct. In our system, the mKate2 signal at 120 h exceeded the limit of detection, defined from the blank of the isogenic EGFP-expressing strain K1, for the RhPV, TEV, and PVY IRES elements, but in none of these constructs did it reach the limit of quantification (Section 2.2; Supplementary File S4, Tables S5 and S6). However, in the case of IRES-directed translation, the amount of mKate2 should change in direct proportion to the amount of the coding mRNA, which was not observed in our experiments: induction of P*AOX1* led to a more than 50-fold increase in the amount of EGFP within just 24 h (Figure 2; Supplementary File S4), whereas the amount of mKate2 after the addition of the inducer remained essentially unchanged for any of the IRES elements tested. Consistently, mKate2 was not detected by immunoblotting in any of the bicistronic strains, either as the full-length protein or as shorter antibody-reactive polypeptides, even upon prolonged exposure (Figure 3A; Supplementary File S2, Figure S3B). Similarly, when constructs with the P*THI11* promoter were used, its inhibition by thiamine was accompanied by a gradual decrease in the amount of EGFP but did not suppress mKate2 synthesis: the constructs with the TEV and the PVY IRES elements behaved in the same way, the mKate2 signal increasing about twofold over the first 24–48 h and thereafter remaining above its initial level and above the limit of quantification until the end of the experiment (Supplementary File S4, Table S8).

The dissociation between the two cistrons was most evident after deletion of the external promoter: the EGFP signal fell to 1.3–2.0% of that in the corresponding P*AOX1*-driven strains, whereas the mKate2 signal was not reduced at all and amounted to 105–178% of its level in the same constructs with an active promoter (Section 2.4; Supplementary File S4, sheet ∆AOX1). Importantly, the level of mKate2 was independent of the level of EGFP both upon induction and upon repression of the promoter, which rules out both coupling of translation of the second cistron to that of the first and any appreciable contribution of spectral overlap of the EGFP signal into the mKate2 detection channel. It may also be noted that a residual mKate2 signal was observed only in the constructs carrying the TEV, PVY, and RhPV inserts, whereas the isogenic cassettes with the EMCV, HCV, and GPR1 inserts, which differ from them only in the inserted sequence, gave a signal that did not exceed that of the blank (Supplementary File S4, Table S6). This is consistent with the residual transcription being determined by the inserted sequence rather than by the flanking regions of the cassette, although it does not identify the element responsible. Taken together, these data indicate that none of the IRES elements tested is able to support efficient internal translation initiation in *K. phaffii*. This underscores the need for careful control of artifacts in the study of IRES-dependent translation.

The sensitivity of the system is defined numerically rather than assumed, which makes it possible to give the negative result a quantitative boundary. The monocistronic construct K2, in which the same mKate2 ORF is expressed cap-dependently from P*AOX1*, provides the upper reference point, and the isogenic EGFP-expressing strain K1 provides the blank; relative to K2, the limit of detection and the limit of quantification correspond to 0.40% and 0.78%, respectively (Section 2.2; Supplementary File S4, Table S5). An element directing internal initiation at even 1% of the cap-dependent expression of the same ORF would therefore have been not merely detected but quantified in this system. Such a threshold is attainable because the green-to-red spectral bleed-through of the reporter pair used here is low; a lower threshold could be reached with a different pair of fluorescent proteins, but it would not alter the conclusion, since an element supporting less than one per cent of cap-dependent expression would be of no practical use for polycistronic expression. The reporter itself is likewise not the factor that limits the outcome: the mKate2 ORF and its 3′ flanking region are identical in all six bicistronic cassettes, the same ORF is efficiently expressed in the monocistronic control, and no shorter antibody-reactive products were found in the bicistronic strains, so that neither impaired maturation nor truncation of the protein can account for the low signal. In the earlier studies, in which IRES activity was recorded through β-galactosidase, the measurements were not accompanied by an explicitly defined detection limit, so that positive and negative outcomes could not be placed on a common quantitative scale.

Two limitations of the present study should be stated explicitly. First, the system includes a positive control for the detection of mKate2, but not for internal initiation as such: no IRES element with independently validated activity in *K. phaffii* is available, since the elements reported to be functional in this yeast originate from the same screens whose conclusions are being re-examined here and have not been confirmed by an orthogonal method. In the absence of such a control, our result has to be stated as a bound rather than as a general property of these elements: in the constructs examined, internal initiation, if it occurs at all, does not exceed the level that the assay can resolve, and weaker activity cannot be excluded. Identifying an element that could serve as such a control is itself a task for which the present system is suited, since it distinguishes IRES-dependent translation from cryptic transcription and places both on a common quantitative scale.

Second, each element was examined in a single context: one architecture of the expression cassette, a single integrated copy at one genomic locus, and one set of cultivation conditions. Cap-independent translation in yeast is known to be manifested in particular physiological states, for example, during starvation-induced differentiation [32], so that the absence of activity under the conditions used here does not exclude it under others. The bicistronic cassette described here can be combined with promoters of different specificity, which makes it possible to test IRES elements over a range of physiological conditions.

One of the practical questions motivating this work was whether the known IRES elements could be used for the construction of polycistronic expression cassettes. The results obtained showed that the elements tested possess weak cryptic promoter activity rather than the ability to initiate cap-independent translation; for RhPV, as noted above, the interpretation is additionally constrained by the loss of the 3′ portion of the transcript, though the practical conclusion is the same, since the amount of second-cistron product is low whichever cause predominates. Moreover, their promoter activity is extremely low and does not exceed 0.9% of the activity of P*AOX1*. Thus, the use of the RhPV, TEV, and PVY IRES elements for the coordinated expression of several genes within polycistronic constructs in *K. phaffii* is not advisable.

## 4. Materials and Methods

### 4.1. Strains

*E. coli* strain DH5α (F^−^, *gyrA96*, (Nal^r^), *recA1*, *relA1*, *endA1*, *thi-1*, *hsdR17*, (r_k_^−^m_k_^+^) *glnV44*, *deoR*, Δ(*lacZYA-argF*) U169 [Φ80dΔ(*lacZ*)M15]) was used as the host for plasmid construction. Analysis of IRES activity was carried out in *K. phaffii* strain GS115.

### 4.2. Culture Media

*E. coli* was cultured in LB medium containing (g/L): peptone, 10; yeast extract, 5; NaCl, 10. Solid LB medium contained 15 g/L agar. Antibiotics were used at the following concentrations (µg/mL): kanamycin, 40; ampicillin, 75 in solid media and 100 in liquid media.

*K. phaffii* was cultured in the following media: YPD (yeast extract, 10 g/L; peptone, 20 g/L; glucose, 20 g/L); YNB agar (minimal medium for clone selection: 1.34% YNB, 10^−5^% biotin, 2% dextrose, 2% agar); and YPM (methanol complex medium: yeast extract, 10 g/L; peptone, 20 g/L; 10^−5^% biotin, 0.5% methanol).

### 4.3. Oligonucleotides and IRES Sequences

The sequences of the oligonucleotides and IRES elements used for plasmid construction are given in Supplementary File S1.

### 4.4. Genetic Constructs with Expression Cassettes

Details of plasmid construction are given in Supplementary File S2.

Plasmid p9KAox-GTK was constructed on the basis of the pPIC9K vector (Invitrogen, Waltham, MA, USA). Under the control of the AOX1 promoter, it contains the EGFP ORF, followed by the TEV IRES and the mKate2 ORF.

Plasmid p9KAox-GPK was constructed on the basis of pPIC9K. Under the control of the AOX1 promoter, it contains the EGFP ORF, followed by the PVY IRES and the mKate2 ORF.

The remaining bicistronic constructs were obtained by replacing the IRES element in p9KAox-GTK. Plasmid p9KAox-GGK contains the *S. cerevisiae* GPR1 IRES; p9KAox-GHK, the HCV IRES; p9KAox-GRK, the RhPV IGR IRES; and p9KAox-GEK, the EMCV IRES.

Plasmid pHik-GTK was derived from p9KAox-GTK by deletion of the AOX1 promoter. Plasmid pHik-GPK was derived from p9KAox-GPK by deletion of the AOX1 promoter. Plasmid pHik-GRK was derived from p9KAox-GRK by deletion of the AOX1 promoter.

Plasmid p9KAox-G is identical to p9KAox-GPK except for a single-nucleotide deletion causing a − 1 frameshift in the mKate2 ORF, which introduces a stop codon at the fifth codon of that ORF. It was selected as a random mutant during the construction of p9KAox-GPK and was used as a control for assessing cap-dependent expression of the green fluorescent protein.

Plasmid p9KAox-K was constructed on the basis of pPIC9K and contains the mKate2 ORF under the control of the AOX1 promoter; it was used as a control for assessing cap-dependent expression of the red fluorescent protein.

Plasmid p9KTh11 was used as an expression vector; it was constructed on the basis of pPIC9K by replacing the AOX1 promoter with THI11.

Plasmid p9KTh11-GTK was constructed on the basis of p9KTh11. Under the control of the THI11 promoter, it contains the EGFP ORF, followed by the TEV IRES and the mKate2 ORF.

Plasmid p9KTh11-GPK was constructed on the basis of p9KTh11. Under the control of the THI11 promoter, it contains the EGFP ORF, followed by the PVY IRES and the mKate2 ORF.

The expression cassettes of all the plasmids listed above were verified by Sanger sequencing.

### 4.5. Screening of Recombinant K. phaffii Clones

Selection of recombinant clones carrying the plasmid region with the genes of interest integrated into the genome was performed on the basis of restoration of histidine prototrophy. For this purpose, after electroporation with the corresponding plasmid, cells were plated on solid YNB medium lacking histidine (YNB agar). Plates were incubated at 30 °C for 2 days.

Three to six preselected transformants were taken for analysis for each construct. Overnight cultures were grown in YPD medium for 16 h at 30 °C. Cells from 1 mL of the overnight culture were then pelleted by centrifugation for 1 min at 5000× *g* and resuspended in 1 mL of YP medium containing the appropriate carbon source (2% methanol for strains with EGFP under the control of the AOX1 promoter and for the promoterless pHik-derived strains, or 2% glucose for strains with EGFP under the control of the THI11 promoter). Cultivation was carried out at 30 °C in a 24-well deep-well plate for 120 h. At each sampling, fresh medium with methanol or glucose was added as a feed.

### 4.6. Verification of the Integrated Expression Cassettes by Nanopore Sequencing

Genomic DNA was isolated from the recombinant *K. phaffii* clones according to the Multi-Copy Pichia Expression Kit protocol (Invitrogen, Waltham, MA, USA), except that the biomass was homogenized by grinding in liquid nitrogen. The chromosomal region spanning the integrated cassette was amplified with the primers SeqAOXpr Up and SeqTerAox lo (Supplementary File S1), which anneal to the AOX1 promoter and terminator, respectively; under these conditions, both the integrated expression cassette and the parental AOX1 locus are amplified. Amplification was carried out with Taq DNA polymerase under standard conditions, with the annealing temperature set according to the melting temperature (Tm) of the primers. One clone of each of the six P*AOX1*-driven constructs (p9KAox-GTK, -GPK, -GGK, -GHK, -GRK, and -GEK) was analyzed. The PCR products were purified with the QIAquick PCR Purification Kit (Qiagen, Hilden, Germany).

Complete sequencing of the PCR fragments was performed by Cloning Facility (Moscow, Russia; https://cloning.tech/) using nanopore sequencing (Oxford Nanopore Technologies, ONT, Oxford, UK). Base-calling was carried out by the service, and the resulting base-called reads (FASTQ) were used for the analysis described below. The reads were mapped to the reference sequences of the expected amplicons—the parental AOX1 locus and the corresponding integrated cassette (Supplementary File S2)—using minimap2 (v2.31) [35]. For each construct, read coverage across the cassette was assessed, and a majority-rule consensus was derived from the alignment and compared with the reference sequence.

### 4.7. Real-Time qPCR: Standard Curves and Copy-Number Determination

Real-time PCR for the standard curves and for copy-number determination was carried out on a DTprime 4 real-time thermal cycler (DNA Technology, Moscow, Russia) with the intercalating dye EvaGreen and a ready-to-use qPCR master mix (Syntol, Moscow, Russia). Genomic DNA was isolated as described in Section 4.6. The mKate2, EGFP, and *K. phaffii* GAPDH markers were amplified with the primer pairs RT-mK2 up/RT-mK2 lo, RT-EGFP up/RT-EGFP lo, and RT-GAPDh up/RT-GAPDh lo, respectively (primer sequences in Supplementary File S1). Standard curves for each marker were constructed from four 10-fold serial dilutions of genomic DNA isolated from a recombinant *K. phaffii* strain carrying the expression cassette. All assays showed high linearity (R^2^ ≥ 0.99) and amplification efficiencies within the acceptable range (104.9–113.1%; GAPDH 105.4%, mKate2 104.9%, EGFP 113.1%), confirming that the curves were suitable for relative quantification (Supplementary File S3, Table S2). Amplification specificity was verified by melt-curve (dissociation) analysis, which gave a single product of the expected size for each primer pair.

The number of integrated cassette copies was determined by the standard-curve method. The Cp of each marker (mKate2, EGFP) was converted to a relative DNA quantity using the corresponding standard curve, and the value was normalized to that of the single-copy reference gene GAPDH; the resulting marker/GAPDH ratio approximates the number of integrated copies per haploid genome. Untransformed strain GS115 served as a specificity control. Measurements were performed in triplicate.

### 4.8. Analysis of Fluorescent K. phaffii Cells

For fluorescence analysis, cells were pelleted by centrifugation at 5000× *g* for 1 min. The supernatant was removed, and the pellet was resuspended in phosphate-buffered saline (PBS). The resulting cell suspension was brought to a volume of 1000 µL (concentrated suspension). For subsequent analysis, 100 µL of this suspension was transferred to a separate tube and 9 volumes of PBS were added, thus yielding a diluted cell suspension. The diluted samples were used for fluorescence detection on a CytoFLEX S flow cytometer (Beckman Coulter, Brea, CA, USA). Analysis was performed for 10^5^ events. For detection of the green EGFP fluorescence signal, a 488 nm laser and a 525/40 nm filter (FITC channel) were used. For detection of the red mKate2 fluorescence signal, a 561 nm laser and a 610/20 nm filter (mCherry channel) were used.

The limit of detection (LOD) and limit of quantification (LOQ) of the mKate2 signal were estimated using the blank-based (signal-to-noise) approach [36,37] as LOD = $\overset{-}{X}$_bl + 3·S_bl and LOQ = $\overset{-}{X}$_bl + 10·S_bl, where $\overset{-}{X}$_bl and S_bl are the mean and the standard deviation of the blank, respectively. The blank was defined as the mCherry-channel signal of strain K1, an isogenic EGFP-expressing control carrying a frameshifted mKate2 ORF (no functional mKate2), which reflects green-to-red spectral bleed-through. This gave LOD = 2163 RFU and LOQ = 4207 RFU (*n* = 3, at 120 h post-induction). Autofluorescence of the host strain GS115, cultivated under the same conditions as the corresponding recombinant strains, was subtracted from all fluorescence values prior to analysis.

### 4.9. Western Blot and In-Gel Fluorescence Analysis of Protein Production from the Constructs Under the Control of the P_AOX1_ Promoter

Expression of the fluorescent protein genes was carried out as described above. At 48 h after induction, *K. phaffii* cells from 2 mL of culture were collected by centrifugation at 5000× *g* for 10 min at 4 °C. The cells were disrupted by grinding the biomass in liquid nitrogen. The resulting paste was resuspended in 300 µL of buffer (25 mM Tris-HCl, 150 mM NaCl, 1 mM EDTA, 1% NP-40, pH 7.4). Cell debris was removed by centrifugation at 10,000× *g* for 10 min at 4 °C. The protein concentration in the clarified lysate was determined from the absorbance at 280 nm (A280). Clarified lysates (18 µg of total protein per lane) were separated by 12% SDS-PAGE, with the PageRuler™ Plus Prestained Protein Ladder (10–250 kDa, Thermo Scientific, Waltham, MA, USA) as the molecular weight marker, and were analyzed by Western blotting as described [38]. After transfer, the membrane was stained with Ponceau S (Supplementary File S2, Figure S3A) before blocking. mKate2 was detected with rabbit polyclonal anti-mKate2 antibodies (Anti-tRFP, cat. #AB233, Evrogen, Moscow, Russia) at a dilution of 1:2000, followed by an HRP-conjugated goat anti-rabbit IgG polyclonal antibody (Cloud-Clone) as the secondary antibody at a dilution of 1:5000. HRP activity was detected using the SuperSignal West Pico PLUS chemiluminescent substrate (Thermo Scientific).

For detection of EGFP fluorescence, clarified lysates (18 µg of total protein per lane, as described above) were separated by 12% SDS-free (non-denaturing) PAGE and analyzed as described [39]. The gels were run in duplicate: one gel was stained with Coomassie G-250 as a loading control, and the other was used for in-gel fluorescence visualization on a Typhoon FLA 9500 imager (GE Healthcare, Chicago, IL, USA).

### 4.10. RNA Isolation and Reverse-Transcription qPCR

Total RNA was isolated from *K. phaffii* cells 48 h after induction. Two millilitres of the induced culture were centrifuged at 5000× *g* for 10 min at 4 °C, and the cells were disrupted by grinding the biomass in liquid nitrogen. RNA was then extracted with the RNA Solo kit (Evrogen, Moscow, Russia) according to the manufacturer’s protocol. First-strand cDNA synthesis and quantitative PCR were performed in a single combined reverse-transcription–PCR reaction using a commercial RT-PCR kit (Alfa-Ferment, Moscow, Russia) supplemented with 50× EvaGreen solution (Syntol, Moscow, Russia) to a final concentration of 1×, on the DTprime 4 real-time thermal cycler. Reverse transcription was primed gene-specifically by the reverse (lower-strand) primer of each marker pair and carried out at 50 °C to minimize the influence of RNA secondary structure. The EGFP (5′ cistron) and mKate2 (3′ cistron) markers were amplified with the RT-EGFP and RT-mK2 primer pairs (Supplementary File S1), the same pairs as used for the standard curves (Section 4.7); for each marker, the reverse primer annealed within the 3′-terminal (stop-codon) region of the corresponding ORF. Transcript levels were quantified relative to those standard curves (Supplementary File S3, Table S2). Amplification specificity was verified by melt-curve (dissociation) analysis.

### 4.11. In Silico Analysis of the IRES Sequences

The IRES insert sequences were screened in silico for cryptic *S. cerevisiae*-type RNA-processing signals using pattern searches against published consensus motifs. All six inserts were searched for the splicing signals, that is, the 5′ splice site (GUAUGU/GUAAGU) and the branch point (UACUAAC) [40]; up to one mismatch was allowed for the branch-point motif. The RhPV insert was, in addition, searched for the polyadenylation and termination signals, that is, the UA-rich efficiency element (UAUAUA-type) together with the downstream A-rich positioning element [41]; for these signals, the search was extended downstream of the insert into the 5′ portion of the mKate2 ORF, so as to include positioning elements located outside the insert. The AT content was calculated over the entire RhPV insert.

### 4.12. Use of Generative AI

During the preparation of this manuscript, the authors used Claude (Anthropic, San Francisco, CA, USA) [Opus 4.8] to translate the text from Russian into English and to edit its English wording, to verify the internal consistency of the numerical values reported in the text against the primary data files, and to reproduce the pattern searches described in Section 4.11. All outputs were verified by the authors against the primary data, and the authors take full responsibility for the content of this publication. The tool was not used for study design, for data collection, or for the interpretation of the results.

The graphical abstract and Figure 3 and Figures S1–S4 were created with BioRender.com.

## 5. Conclusions

We have proposed a system for screening IRES elements in the yeast *K. phaffii* based on the coordinated expression of two reporter genes within bicistronic EGFP-IRES-mKate2 constructs. The system makes it possible, by flow cytometry, to monitor the expression of both cistrons over time, to verify that the second cistron is retained within the transcript, and, crucially, to distinguish IRES-dependent translation from cryptic promoter activity of the sequences preceding the mKate2 ORF. Since the same mKate2 ORF, expressed cap-dependently from a monocistronic cassette, serves as the reference point, the activity of an IRES element is measured on a common quantitative scale with an explicitly defined limit of detection, which in the present work corresponded to 0.4% of the cap-dependent expression of that ORF.

Under the conditions used here, none of the IRES elements studied, namely the viral EMCV, HCV, TEV, RhPV, and PVY elements and the IRES element of the *S. cerevisiae* GPR1 mRNA, directed detectable cap-independent initiation of translation of the second cistron in *K. phaffii*; activity below the detection limit of the assay cannot be excluded. The mKate2 signal observed for the TEV, PVY, and RhPV IRES elements is apparently due to weak cryptic promoter activity of the sequences preceding the mKate2 ORF rather than to an IRES mechanism as such. For the construct carrying the RhPV intergenic region, the interpretation is additionally constrained by the specific depletion of the 3′ portion of the transcript, and our conclusion for this element is correspondingly more cautious: with the template reduced approximately 16-fold, the detection threshold for this construct corresponds to about 6%, rather than 0.4%, of the cap-dependent expression of the same ORF.

The approach reported here provides the first quantitative estimate of the activity of these elements in *K. phaffii* and, more generally, a way of testing candidate IRES elements without mistaking cryptic transcription for internal initiation. Since the same cassette can be placed under the control of promoters of different specificity, and since the sensitivity of the assay can be adjusted through the choice of the reporter pair, the system can serve as a screening tool in the search for elements that do support cap-independent translation in this yeast—a search that would supply functional IRES elements for both fundamental studies and biotechnological applications.

## Figures and Tables

**Table 1:** Relative abundance of the 5′ (EGFP) and 3′ (mKate2) regions within the bicistronic transcript by RT-qPCR in EGFP–IRES–mKate2 strains.

Construct (IRES)	Cp EGFP (5′ Cistron)	Cp mKate2 (3′ Cistron)	ΔCp (3′ − 5′)	no-RT Cp EGFP	no-RT Cp mKate2
TEV	12.5	13.2	0.7 ± 0.3	27.4	28.8
PVY	11.7	12.6	0.9 ± 0.5	27.0	28.2
EMCV	11.5	12.5	1.0 ± 0.8	26.9	28.5
HCV	11.5	12.6	1.1 ± 0.2	26.4	29.2
GPR1	12.2	13.9	1.7 ± 0.1	27.5	29.0
RhPV	11.6	16.7	5.1 ± 0.2	25.8	28.0

Total RNA was isolated 48 h after induction. Both primer pairs target the 3′-terminal (stop-codon) region of the respective ORF; ΔCp = Cp (mKate2)—Cp (EGFP) reports the intramolecular 3′/5′ imbalance within the same transcript. Values are means of *n* = 3. Control reactions performed without reverse transcriptase (no-RT) gave Cp values 11.2–16.6 cycles higher than the corresponding complete reactions, that is, a more than 2 × 10^3^-fold lower contribution, confirming that the signal originated from RNA; a no-template control gave Cp > 25 for both markers.

**Table 2:** Relative level of mKate2 (second-cistron) expression in the promoterless EGFP-IRES-mKate2 constructs.

IRES	Relative Expression Level of the Second Cistron *, %
RhPV	0.5 ± 0.07
PVY	0.9 ± 0.19
TEV	0.7 ± 0.10

* Calculated for each promoterless construct as the ratio of the mKate2 RFU within the EGFP-IRES-mKate2 cassette to the mKate2 RFU of the monocistronic construct p9KAox-K under the control of PAOX1 (Section 4). Numerical RFU values for mKate2 are given in Supplementary File S4 (sheet RELSC).

## Data Availability

The original contributions presented in this study are included in the article/Supplementary Materials. Further inquiries can be directed to the corresponding author.

## Supplementary Materials

The following supporting information can be downloaded at: https://www.mdpi.com/article/10.3390/ijms27188166/s1.

## Abbreviations

The following abbreviations are used in this manuscript:

IRES	Internal Ribosome Entry Site
EMCV	Encephalomyocarditis virus
HCV	Hepatitis C virus
TEV	Tobacco etch virus
PVY	Potato virus Y
RhPV	Rhopalosiphum padi virus
TRV	Triatoma virus
KSHV	Kaposi-sarcoma-associated herpesvirus
crTMV	Crucifer-infecting Tobacco mosaic virus
5′ UTR	5′ Untranslated Region
GPR1	G-protein-coupled receptor gene
AOX1	Alcohol oxidase 1 gene
THI11	Thiamine biosynthetic gene THI11
GAPDH	Glyceraldehyde-3-phosphate dehydrogenase gene
EGFP	Enhanced green fluorescent protein
ORF	Open reading frame
IGR	Intergenic region
ITAF	IRES trans-acting factor
ONT	Oxford Nanopore Technologies
LOD	Limit of detection
LOQ	Limit of quantification
RFU	Relative fluorescence units
Cp	Crossing point (quantification cycle)
