# Supplementary material for: Reassessment of IRES Element Activity in Komagataella phaffii Using a Standardized Bicistronic System: Cryptic Promoter Activity Mimics Cap-Independent Translation
Source: Int J Mol Sci. 2026 Sep 14;27(18):8166. doi: 10.3390/ijms27188166 (PMC13607543; doi:10.3390/ijms27188166)
Supplement: Supplementary file 1 [file ijms-27-08166-s001.zip › Supplementary file S2.pdf]

Plasmid vectors constructed in this work

p9KAox-GTK

This plasmid contains, under the control of the AOX1 promoter, the EGFP ORF followed by the TEV IRES and the mKate2 ORF. To construct it, the EGFP ORF was amplified from the template pEGFP-N1 with the primers (GFPgrib up; G1T lo and G2T lo), and the target PCR fragment was 810 bp long. The mKate2 ORF was amplified from the template pmKate2 with the primers (KatGrib lo; Kat1T up and Kat2T up), and the target PCR fragment was 781 bp long. The fragment containing the TEV IRES was obtained by annealing the primers (GMostT-Kat and KatMostT-G). The three resulting fragments were assembled into a single contig 1621 bp long by PCR with the primers (GFPgrib up and Katgrib lo). The resulting PCR fragment was cloned into the plasmid vector pPIC9K at the BamHI and NotI sites.

p9KAox-GPK

This plasmid contains, under the control of the AOX1 promoter, the EGFP ORF followed by the PVY IRES and the mKate2 ORF. To construct it, the EGFP ORF was amplified from the template pEGFP-N1 with the primers (GFPgrib up; G1P lo and G2P lo), and the target PCR fragment was 820 bp long. The mKate2 ORF was amplified from the template pmKate2 with the primers (KatGrib lo; Kat1P up and Kat2P up), and the target PCR fragment was 802 bp long. The fragment containing the PVY IRES was obtained by annealing the primers (GMostP-Kat and KatMostP-G). The three resulting fragments were assembled into a single contig 1662 bp long by PCR with the primers (GFPgrib up and Katgrib lo). The resulting PCR fragment was cloned into the plasmid vector pPIC9K at the BamHI and NotI sites.

p9KAox-GGK

This plasmid contains, under the control of the AOX1 promoter, the EGFP ORF followed by the GPR1 IRES and the mKate2 ORF. A DNA fragment containing the IRES sequence of the GPR1 gene, 434 bp long, was amplified by PCR from the template pPICZαGPR (kindly provided by Irina Pozdnyakova-Filatova) using the primers gpr1 up and gpr1 lo. The mKate2 ORF (722 bp) was amplified by PCR from the template p9KAox-GTK with the primers g-Kate up and KatGrib lo. The resulting fragments were assembled into a single contig 1133 bp long by PCR with the primers gpr1 up and KatGrib lo. The resulting PCR fragment was cloned into plasmid p9KAox-GTK at the EcoRI and NotI sites.

p9KAox-GHK

This plasmid contains, under the control of the AOX1 promoter, the EGFP ORF followed by the HCV IRES and the mKate2 ORF. A DNA fragment containing the HCV IRES, 400 bp long, was amplified by PCR from the HCV IRES template (the sequence is given in Supplementary file 1) using the primers hcv up and hcv lo. The mKate2 ORF (730 bp) was amplified by PCR from the template p9KAox-GTK with the primers h-Kate up and KatGrib lo. The resulting fragments were assembled into a single contig 1104 bp long by PCR with the primers hcv up and KatGrib lo. The resulting PCR fragment was cloned into plasmid p9KAox-GTK at the EcoRI and NotI sites.

p9KAox-GRK

This plasmid contains, under the control of the AOX1 promoter, the EGFP ORF followed by the RhPV IRES and the mKate2 ORF. A DNA fragment containing the RhPV IRES, 570 bp long, was amplified by PCR from the RhPV IRES template (the sequence is given in Supplementary file 1) using the primers rhpv up and rhpv lo. The mKate2 ORF (722 bp) was amplified by PCR from the template p9KAox-GTK with the primers r-Kate up and KatGrib lo. The resulting fragments were assembled into a single contig 1270 bp long by PCR with the primers rhpv up and KatGrib lo. The resulting PCR fragment was cloned into plasmid p9KAox-GTK at the EcoRI and NotI sites.

p9KAox-GEK

This plasmid contains, under the control of the AOX1 promoter, the EGFP ORF followed by the EMCV IRES and the mKate2 ORF. A DNA fragment containing the EMCV IRES, 619 bp long, was amplified by PCR from the template pTRIPZ (M)-HT-Cbx4 (gift from Xiaojun Ren (Addgene plasmid #82513; http://n2t.net/addgene:82513; RRID: Addgene 82513)) using the primers emcv up and emcv lo. The mKate2 ORF (724 bp) was amplified by PCR from the template p9KAox-GTK with the primers r-Kate up and KatGrib lo. The resulting fragments were assembled into a single contig 1319 bp long by PCR with the primers emcv up and KatGrib lo. The resulting PCR fragment was cloned into plasmid p9KAox-GTK at the EcoRI and NotI sites.

pHik-GTK, pHik-GPK, and pHik-GRhK

Plasmid pHik-GTK is analogous to p9KAox-GTK except that the AOX promoter has been deleted from the vector. The plasmid was derived from p9KAox-GTK. To construct the vector,

p9KAox-GTK was treated with the restriction enzymes BamHI, BglII, and SacI, with complete digestion at the BamHI and SacI sites and partial digestion at the BglII site, and was ligated to itself. Constructs pHik-GPK and pHik-GRK were obtained in the same way from the parental plasmids p9KAox-GPK and p9KAox-GRK, respectively.

p9KAox-K

This plasmid contains the mKate2 ORF under the control of the AOX1 promoter. The mKate2 ORF (728 bp) was amplified by PCR from the template p9KAox-GTK with the primers KatGrib lo and BamKat up. The resulting PCR fragment was cloned into plasmid pPIC9K at the BamHI and NotI sites.

p9KAox-G

This plasmid contains the EGFP ORF under the control of the AOX1 promoter. It was randomly selected during the construction of p9KAox-GPK. It contains a single-nucleotide deletion that converts the fifth codon of the mKate2 ORF into a stop codon.

p9KTh11

This plasmid is analogous to pPIC9K except that it contains the THI11 promoter instead of the AOX1 promoter. To construct it, a DNA fragment of *P. pastoris* GS115, 1023 bp long, containing the THI11 promoter, was amplified by PCR using the primers THI11 up and THI11 lo. The resulting PCR fragment was cloned into plasmid pPIC9K at the BamHI and AatII sites.

p9KTh11-GPK

This plasmid contains, under the control of the THI11 promoter, the EGFP ORF followed by the PVY IRES and the mKate2 ORF. It was derived from the vector p9KTh11 by cloning the 1657 bp BamHI/NotI fragment of plasmid p9KAox-GPK, which contains the EGFP-IRES-mKate2 cassette (with the PVY IRES), at the corresponding sites.

p9KTh11-GTK

This plasmid contains, under the control of the THI11 promoter, the EGFP ORF followed by the TEV IRES and the mKate2 ORF. It was derived from the vector p9KTh11 by cloning the 1616 bp BamHI/NotI fragment of plasmid p9KAox-GTK, which contains the EGFP-IRES-mKate2 cassette (with the TEV IRES), at the corresponding sites.

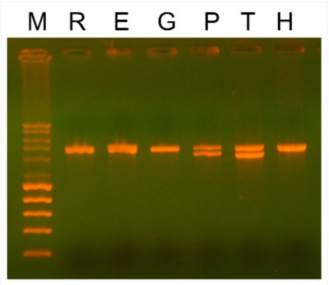

Figure S1. PCR amplification of genomic DNA fragments with the primers SeqAOXpr Up and SeqTerAox lo from the K. phaffii GS115 recombinant strains. Lanes: M, DNA ladder (5, 4, 3, 2.5, 2, 1.5, 1.25, 1, 0.8, 0.6, and 0.4 kb); R, E, G, P, T, H, recombinant strains carrying the bicistronic cassettes with the RhPV, EMCV, GPR1, PVY, TEV, and HCV IRES elements, respectively. In each lane, the two closely migrating bands correspond to the co-amplified integrated cassette and the parental AOX1 locus.

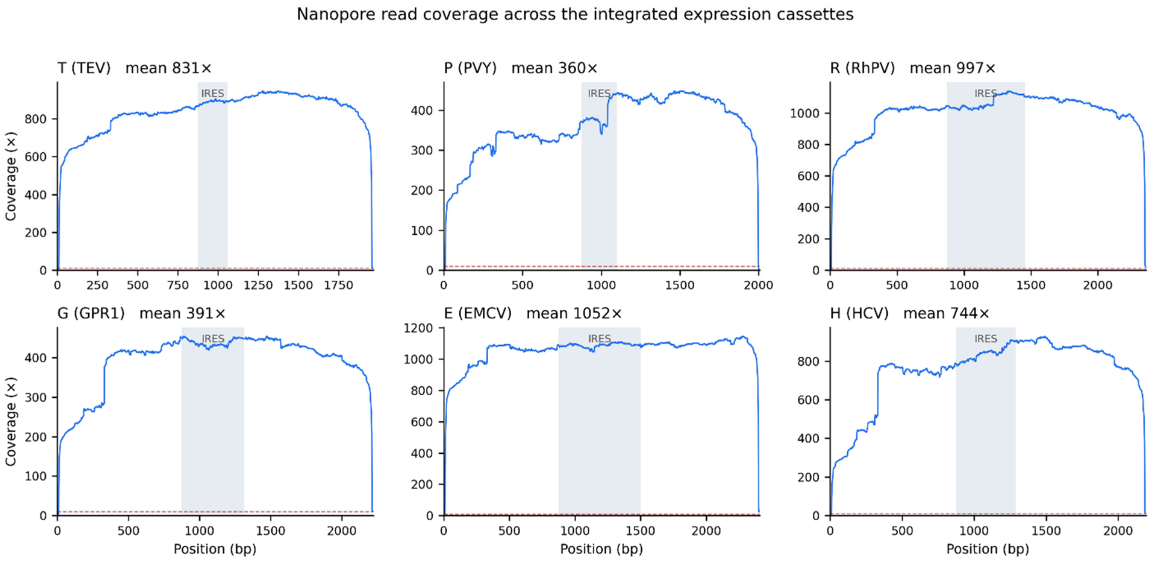

Figure S2. Nanopore read coverage across the integrated expression cassettes. For each of the six PAOX1-driven constructs (T, TEV; P, PVY; R, RhPV; G, GPR1; E, EMCV; H, HCV), the coverage distribution along the integrated-cassette sequence is shown: the x-axis is the position (bp) and the y-axis is the coverage (number of reads). Reads were obtained by sequencing the genomic PCR fragments and mapped to the designed cassette sequences (Materials and Methods; Supplementary file 2). The IRES region is shaded, the dashed line marks the 10× coverage level, and the mean coverage is indicated above each panel.

Table S4. Nanopore sequencing verification of the integrated expression cassettes.

Construct (IRES)	Total ONT reads	Reads: cassette	Reads: AOX1 locus	Non-specific reads, %	Mean coverage, ×	Cassette at ≥10×, %	Consensus identity, %
**T (TEV)**	4004	2057	1794	3.8	831	99.2	100.00
**P (PVY)**	2373	1155	921	12.5	360	99.1	100.00
**R (RhPV)**	6415	2710	3396	4.8	997	99.7	100.00
**G (GPR1)**	2418	1019	1183	8.9	391	99.6	99.96 †
**E (EMCV)**	6772	2776	3704	4.3	1052	99.6	100.00
**H (HCV)**	5371	2282	2723	6.8	744	99.4	100.00

Genomic DNA amplicons (AOX1 promoter–cassette–AOX1 terminator) co-amplify the integrated cassette and the parental AOX1 locus; "non-specific reads" are reads not mapping to either expected fragment. Mean coverage, coverage breadth and consensus identity are given for the integrated cassette.

† The single discrepant position lies within a homopolymeric A-tract of the GPR1 IRES and reflects the known limitation of nanopore sequencing in resolving homopolymer length; the sequence of this region was confirmed by Sanger sequencing of the plasmid.

Sequences of the genomic DNA fragments amplified with the primers SeqAOXpr Up and SeqTerAox lo from the K. phaffii GS115 recombinant strains.

AOX1 locus of K. phaffii GS115, 2272 bp

CAGAAGGAAGCTGCCCTGTCTTAAACCTTTTTTTTTATCATCATTATTAGCTTACTTTCATAATTGCGACTGGTT
CCAATTGACAAGCTTTTGATTTTAACGACTTTTAACGACAACTTGAGAAGATCAAAAAACAACTAATTATTCGAA
ACGATGGCTATCCCCGAAGAGTTTGATATCCTAGTTCTAGGTGGTGGATCCAGTGGATCCTGTATTGCCGGAAGA
TTGGCAAACTTGGACCACTCCTTGAAAGTTGGTCTTATCGAAGCAGGTGAGAACAACCTCAACAACCCATGGGTC
TACCTTCCAGGTATTTACCCAAGAAACATGAAGTTGGACTCCAAGACTGCTTCCTTCTACACTTCTAACCCATCT
CCTCACTTGAATGGTAGAAGAGCCATTGTTCCATGTGCTAACGTCTTGGGTGGTGGTTCTTCTATCAACTTCATG
ATGTACACCAGAGGTTCTGCTTCTGATTACGATGACTTCCAAGCCGAGGGCTGGAAAACCAAGGACTTGCTTCCA
TTGATGAAAAAGACTGAGACCTACCAAAGAGCTTGCAACAACCCTGACATTCACGGTTTCGAAGGTCCAATCAAG
GTTTCTTTCGGTAACTACACCTACCCAGTTTGCCAGGACTTCTTGAGGGCTTCTGAGTCCCAAGGTATTCCATAC
GTTGACGACTTGGAAGACTTGGTTACTGCTCACGGTGCTGAACACTGGTTGAAGTGGATCAACAGAGACACTGGT
CGTCGTTCCGACTCTGCTCATGCATTTGTCCACTCTACTATGAGAAACCACGACAACTTGTACTTGATCTGTAAC
ACGAAGGTCGACAAAATTATTGTCGAAGACGGAAGAGCTGCTGCTGTTAGAACCGTTCCAAGCAAGCCTTTGAAC
CCAAAGAAGCCAAGTCACAAGATCTACCGTGCTAGAAAGCAAATCGTTTTGTCTTGTGGTACCATCTCCTCTCCA
TTGGTTTTGCAAAGATCCGGTTTTGGTGACCCAATCAAGTTGAGAGCCGCTGGTGTTAAGCCTTTGGTCAACTTG
CCAGGTGTCGGAAGAAACTTCCAAGACCACTACTGTTTCTTCAGTCCTTACAGAATCAAGCCTCAGTACGAGTCT
TTCGATGACTTCGTCCGTGGTGATGCTGAGATTCAAAAGAGAGTCTTTGACCAATGGTACGCCAATGGTACTGGT
CCTCTTGCCACTAACGGTATCGAAGCTGGTGTCAAGATCAGACCAACACCAGAAGAACTCTCTCAAATGGACGAA
TCCTTCCAGGAGGGTTACAGAGAATACTTCGAAGACAAGCCAGACAAGCCAGTTATGCACTACTCCATCATTGCT
GGTTTCTTCGGTGACCACACCAAGATTCCTCCTGGAAAGTACATGACTATGTTCCACTTCTTGGAATACCCATTC
TCCAGAGGTTCCATTCACATTACCTCCCCAGACCCATACGCAGCTCCAGACTTCGACCCAGGTTTCATGAACGAT
GAAAGAGACATGGCTCCTATGGTTTGGGCTTACAAGAAGTCTAGAGAAACCGCTAGAAGAATGGACCACTTTGCC
GGTGAGGTCACTTCTCACCACCCTCTGTTCCCATACTCATCCGAGGCCAGAGCCTTGGAAATGGATTTGGAGACC
TCTAATGCCTACGGTGGACCTTTGAACTTGTCTGCTGGTCTTGCTCACGGTTCTTGGACTCAACCTTTGAAGAAG
CCAACTGCAAAGAACGAAGGCCACGTTACTTCGAACCAGGTCGAGCTTCATCCAGACATCGAGTACGATGAGGAG
GATGACAAGGCCATTGAGAACTACATTCGTGAGCACACTGAGACCACATGGCACTGTCTGGGAACCTGTTCCATC
GGTCCAAGAGAAGGTTCCAAGATCGTCAAATGGGGTGGTGTTTTGGACCACAGATCCAACGTTTACGGAGTCAAG
GGCTTGAAGGTTGGTGACTTGTCCGTGTGCCCAGACAATGTTGGTTGTAACACCTACACCACCGCTCTTTTGATC
GGTGAAAAGACTGCCACTTTGGTTGGAGAAGATTTAGGATACTCTGGTGAGGCCTTAGACATGACTGTTCCTCAG
TTCAAGTTGGGCACTTACGAGAAGACCGGTCTTGCTAGATTCTAATCAAGAGGATGTCAGAATGCCATTTGCCTG
AGAGATGCAGGCTTCATTTTTGATTACTTTTTTATTTGTAACCTATATAGTATAGGATTTTTTTTGTCATTTTGT
TTCTTCTCGTACGAGCTTGCTC

Sequence of the expression cassette of p9KAox-GGK (2223 bp)

CAGAAGGAAGCTGCCCTGTCTTAAACCTTTTTTTTTATCATCATTATTAGCTTACTTTCATAATTGCGA
CTGGTTCCAATTGACAAGCTTTTGATTTTAACGACTTTTAACGACAACTTGAGAAGATCAAAAAACAACTAATTA
TTCGAAGGATCCAAACGATGGTGAGCAAGGGCGAGGAGCTGTTCACCGGGGTGGTGCCCATCCTGGTCGAGCTGG
ACGGCGACGTAAACGGCCACAAGTTCAGCGTGTCCGGCGAGGGCGAGGGCGATGCCACCTACGGCAAGCTGACCC
TGAAGTTCATCTGCACCACCGGCAAGCTGCCCGTGCCCTGGCCCACCCTCGTGACCACCCTGACCTACGGCGTGC
AGTGCTTCAGCCGCTACCCCGACCACATGAAGCAGCACGACTTCTTCAAGTCCGCCATGCCCGAAGGCTACGTCC
AGGAGCGCACCATCTTCTTCAAGGACGACGGCAACTACAAGACCCGCGCCGAGGTGAAGTTCGAGGGCGACACCC
TGGTGAACCGCATCGAGCTGAAGGGCATCGACTTCAAGGAGGACGGCAACATCCTGGGGCACAAGCTGGAGTACA
ACTACAACAGCCACAACGTCTATATCATGGCCGACAAGCAGAAGAACGGCATCAAGGTGAACTTCAAGATCCGCC
ACAACATCGAGGACGGCAGCGTGCAGCTCGCCGACCACTACCAGCAGAACACCCCCATCGGCGACGGCCCCGTGC
TGCTGCCCGACAACCACTACCTGAGCACCCAGTCCGCCCTGAGCAAAGACCCCAACGAGAAGCGCGATCACATGG
TCCTGCTGGAGTTCGTGACCGCCGCCGGGATCACTCTCGGCATGGACGAGCTGTACAAGTGAAGATCCAGTACTG
CAGAATTCAGCTCAGGTGCCCTCTTGTTTTTCTTTTTCATTTTTTTAAATCACACTTTTTTTTCTTTTCAAGATT
TATAAATCTTCCTTCCAGGTTTGAGCCGGACCCATTGTTTAATTAAAGCATAGAAGGCAACGAAGGAGTGGGTTA
TAGAATTAGCATTGCGGCAAAAACGTCATATTTATTAATATTTATTTGCGAAGGTCATATTCAACGCAATCGCAC
GTCCTCTTCCGTCTTTTTTCTAGCTTTTGTCCTTTTTTATTTGTCTACATCCCTTTCTCTACGGTATCCAGAAGA
AGGTGTGTGTGTGTGTGTGTGTGTGTGTCTATAAAAAGCAGTAAGAGTCACCAAAAAAAAAAAACGACAAACAAG
TGATCCGAAGTGTGACGAATAAAGCAAACTCTCCAACTCAAAATGGTGAGCGAGCTGATTAAGGAGAACATGCAC
ATGAAGCTGTACATGGAGGGCACCGTGAACAACCACCACTTCAAGTGCACATCCGAGGGCGAAGGCAAGCCCTAC
GAGGGCACCCAGACCATGAGAATCAAGGCGGTCGAGGGCGGCCCTCTCCCCTTCGCCTTCGACATCCTGGCTACC
AGCTTCATGTACGGCAGCAAAACCTTCATCAACCACACCCAGGGCATCCCCGACTTCTTTAAGCAGTCCTTCCCC
GAGGGCTTCACATGGGAGAGAGTCACCACATACGAAGACGGGGGCGTGCTGACCGCTACCCAGGACACCAGCCTC
CAGGACGGCTGCCTCATCTACAACGTCAAGATCAGAGGGGTGAACTTCCCATCCAACGGCCCTGTGATGCAGAAG
AAAACACTCGGCTGGGAGGCCTCCACCGAGACCCTGTACCCCGCTGACGGCGGCCTGGAAGGCAGAGCCGACATG

GCCCTGAAGCTCGTGGGCGGGGGCCACCTGATCTGCAACTTGAAGACCACATACAGATCCAAGAAACCCGCTAAG
AACCTCAAGATGCCCGGCGTCTACTATGTGGACAGAAGACTGGAAAGAATCAAGGAGGCCGACAAAGAGACCTAC
GTCGAGCAGCACGAGGTGGCTGTGGCCAGATACTGCGACCTCCCTAGCAAACTGGGGCACAGATAAGCGGCCGCG
AATTAATTCGCCTTAGACATGACTGTTCCTCAGTTCAAGTTGGGCACTTACGAGAAGACCGGTCTTGCTAGATTC
TAATCAAGAGGATGTCAGAATGCCATTTGCCTGAGAGATGCAGGCTTCATTTTTGATACTTTTTTATTTGTAACC
TATATAGTATAGGATTTTTTTTGTCATTTTGTTTCTTCTCGTACGAGCTTGCTC

Sequence of the expression cassette of p9KAox-GPK (2007 bp)

CAGAAGGAAGCTGCCCTGTCTTAAACCTTTTTTTTTATCATCATTATTAGCTTACTTTCATAATTGCGACTGGTT
CCAATTGACAAGCTTTTGATTTTAACGACTTTTAACGACAACTTGAGAAGATCAAAAAACAACTAATTATTCGAA
GGATCCAAACGATGGTGAGCAAGGGCGAGGAGCTGTTCACCGGGGTGGTGCCCATCCTGGTCGAGCTGGACGGCG
ACGTAAACGGCCACAAGTTCAGCGTGTCCGGCGAGGGCGAGGGCGATGCCACCTACGGCAAGCTGACCCTGAAGT
TCATCTGCACCACCGGCAAGCTGCCCGTGCCCTGGCCCACCCTCGTGACCACCCTGACCTACGGCGTGCAGTGCT
TCAGCCGCTACCCCGACCACATGAAGCAGCACGACTTCTTCAAGTCCGCCATGCCCGAAGGCTACGTCCAGGAGC
GCACCATCTTCTTCAAGGACGACGGCAACTACAAGACCCGCGCCGAGGTGAAGTTCGAGGGCGACACTCTGGTGA
ACCGCATCGAGCTGAAGGGCATCGACTTCAAGGAGGACGGCAACATCCTGGGGCACAAGCTGGAGTACAACTACA
ACAGCCACAACGTCTATATCATGGCCGACAAGCAGAAGAACGGCATCAAGGTGAACTTCAAGATCCGCCACAACA
TCGAGGACGGCAGCGTGCAGCTCGCCGACCACTACCAGCAGAACACCCCCATCGGCGACGGCCCCGTGCTGCTGC
CCGACAACCACTACCTGAGCACCCAGTCCGCCCTGAGCAAAGACCCCAACGAGAAGCGCGATCACATGGTCCTGC
TGGAGTTCGTGACCGCCGCCGGGATCACTCTCGGCATGGACGAGCTGTACAAGTGAAGATCCAGTACTGCAGAAT
TCAGCTCAGGTAATTAAAACAACTCAATACAACATAAGAAAAACAACGCAAAAACACTCATAAACGCTCATTCTC
ACTCAAGCAACTTGCTAAGTTTCAGTTTAAATCATTTCCTTGCAATTCTCTAGAACAATATTGGAAACCATTTCA
ACTCAACAAGCAATTTCATCACTTCCAACCAATTTCAGATCCTCAATGGTGAGCGAGCTGATTAAGGAGAACATG
CACATGAAGCTGTACATGGAGGGCACCGTGAACAACCACCACTTCAAGTGCACATCCGAGGGCGAAGGCAAGCCC
TACGAGGGCACCCAGACCATGAGAATCAAGGCGGTCGAGGGCGGCCCTCTCCCCTTCGCCTTCGACATCCTGGCT
ACCAGCTTCATGTACGGCAGCAAAACCTTCATCAACCACACCCAGGGCATCCCCGACTTCTTTAAGCAGTCCTTC
CCCGAGGGCTTCACATGGGAGAGAGTCACCACATACGAAGACGGGGGCGTGCTGACCGCTACCCAGGACACCAGC
CTCCAGGACGGCTGCCTCATCTACAACGTCAAGATCAGAGGGGTGAACTTCCCATCCAACGGCCCTGTGATGCAG
AAGAAAACACTCGGCTGGGAGGCCTCCACCGAGACCCTGTACCCCGCTGACGGCGGCCTGGAAGGCAGAGCCGAC
ATGGCCCTGAAGCTCGTGGGCGGGGGCCACCTGATCTGCAACTTGAAGACCACATACAGATCCAAGAAACCCGCT
AAGAACCTCAAGATGCCCGGCGTCTACTATGTGGACAGAAGACTGGAAAGAATCAAGGAGGCCGACAAAGAGACC
TACGTCGAGCAGCACGAGGTGGCTGTGGCCAGATACTGCGACCTCCCTAGCAAACTGGGGCACAGATAAGCGGCC
GCGAATTAATTCGCCTTAGACATGACTGTTCCTCAGTTCAAGTTGGGCACTTACGAGAAGACCGGTCTTGCTAGA
TTCTAATCAAGAGGATGTCAGAATGCCATTTGCCTGAGAGATGCAGGCTTCATTTTTGATACTTTTTTATTTGTA
ACCTATATAGTATAGGATTTTTTTTGTCATTTTGTTTCTTCTCGTACGAGCTTGCTC

Sequence of the expression cassette of p9KAox-GHK (2194 bp)

CAGAAGGAAGCTGCCCTGTCTTAAACCTTTTTTTTTATCATCATTATTAGCTTACTTTCATAATTGCGACTGGTT
CCAATTGACAAGCTTTTGATTTTAACGACTTTTAACGACAACTTGAGAAGATCAAAAAACAACTAATTATTCGAA
GGATCCAAACGATGGTGAGCAAGGGCGAGGAGCTGTTCACCGGGGTGGTGCCCATCCTGGTCGAGCTGGACGGCG
ACGTAAACGGCCACAAGTTCAGCGTGTCCGGCGAGGGCGAGGGCGATGCCACCTACGGCAAGCTGACCCTGAAGT
TCATCTGCACCACCGGCAAGCTGCCCGTGCCCTGGCCCACCCTCGTGACCACCCTGACCTACGGCGTGCAGTGCT
TCAGCCGCTACCCCGACCACATGAAGCAGCACGACTTCTTCAAGTCCGCCATGCCCGAAGGCTACGTCCAGGAGC
GCACCATCTTCTTCAAGGACGACGGCAACTACAAGACCCGCGCCGAGGTGAAGTTCGAGGGCGACACCCTGGTGA
ACCGCATCGAGCTGAAGGGCATCGACTTCAAGGAGGACGGCAACATCCTGGGGCACAAGCTGGAGTACAACTACA
ACAGCCACAACGTCTATATCATGGCCGACAAGCAGAAGAACGGCATCAAGGTGAACTTCAAGATCCGCCACAACA
TCGAGGACGGCAGCGTGCAGCTCGCCGACCACTACCAGCAGAACACCCCCATCGGCGACGGCCCCGTGCTGCTGC
CCGACAACCACTACCTGAGCACCCAGTCCGCCCTGAGCAAAGACCCCAACGAGAAGCGCGATCACATGGTCCTGC
TGGAGTTCGTGACCGCCGCCGGGATCACTCTCGGCATGGACGAGCTGTACAAGTGAAGATCCAGTACTGCAGAAT
TCAGCTCAGGTGCCAGCCCCCTGATGGGGGCGACACTCCACCATGAATCACTCCCCTGTGAGGAACTACTGTCTT
CACGCAGAAAGCGTCTAGCCATGGCGTTAGTATGAGTGTCGTGCAGCCTCCAGGACCCCCCCTCCCGGGAGAGCC
ATAGTGGTCTGCGGAACCGGTGAGTACACCGGAATTGCCAGGACGACCGGGTCCTTTCTTGGATAAACCCGCTCA
ATGCCTGGAGATTTGGGCGTGCCCCCGCAAGACTGCTAGCCGAGTAGTGTTGGGTCGCGAAAGGCCTTGTGGTAC
TGCCTGATAGGGTGCTTGCGAGTGCCCCGGGAGGTCTCGTAGACCGTGCACCATGAGCACGAATCCTAAACCTCA
AAGAAAAATGGTGAGCGAGCTGATTAAGGAGAACATGCACATGAAGCTGTACATGGAGGGCACCGTGAACAACCA
CCACTTCAAGTGCACATCCGAGGGCGAAGGCAAGCCCTACGAGGGCACCCAGACCATGAGAATCAAGGCGGTCGA
GGGCGGCCCTCTCCCCTTCGCCTTCGACATCCTGGCTACCAGCTTCATGTACGGCAGCAAAACCTTCATCAACCA
CACCCAGGGCATCCCCGACTTCTTTAAGCAGTCCTTCCCCGAGGGCTTCACATGGGAGAGAGTCACCACATACGA
AGACGGGGGCGTGCTGACCGCTACCCAGGACACCAGCCTCCAGGACGGCTGCCTCATCTACAACGTCAAGATCAG
AGGGGTGAACTTCCCATCCAACGGCCCTGTGATGCAGAAGAAAACACTCGGCTGGGAGGCCTCCACCGAGACCCT

GTACCCCGCTGACGGCGGCCTGGAAGGCAGAGCCGACATGGCCCTGAAGCTCGTGGGCGGGGGCCACCTGATCTG
CAACTTGAAGACCACATACAGATCCAAGAAACCCGCTAAGAACCTCAAGATGCCCGGCGTCTACTATGTGGACAG
AAGACTGGAAAGAATCAAGGAGGCCGACAAAGAGACCTACGTCGAGCAGCACGAGGTGGCTGTGGCCAGATACTG
CGACCTCCCTAGCAAACTGGGGCACAGATAAGCGGCCGCGAATTAATTCGCCTTAGACATGACTGTTCCTCAGTT
CAAGTTGGGCACTTACGAGAAGACCGGTCTTGCTAGATTCTAATCAAGAGGATGTCAGAATGCCATTTGCCTGAG
AGATGCAGGCTTCATTTTTGATACTTTTTTATTTGTAACCTATATAGTATAGGATTTTTTTTGTCATTTTGTTTC
TTCTCGTACGAGCTTGCTC

Sequence of the expression cassette of p9KAox-GRK (2360 bp)
CAGAAGGAAGCTGCCCTGTCTTAAACCTTTTTTTTTATCATCATTATTAGCTTACTTTCATA
ATTGCGACTGGTTCCAATTGACAAGCTTTTGATTTTAACGACTTTTAACGACAACTTGAGAA
GATCAAAAAACAACTAATTATTCGAAGGATCCAAACGATGGTGAGCAAGGGCGAGGAGCTGT
TCACCGGGGTGGTGCCCATCCTGGTCGAGCTGGACGGCGACGTAAACGGCCACAAGTTCAGC
GTGTCCGGCGAGGGCGAGGGCGATGCCACCTACGGCAAGCTGACCCTGAAGTTCATCTGCAC
CACCGGCAAGCTGCCCGTGCCCTGGCCCACCCTCGTGACCACCCTGACCTACGGCGTGCAGT
GCTTCAGCCGCTACCCCGACCACATGAAGCAGCACGACTTCTTCAAGTCCGCCATGCCCGAA
GGCTACGTCCAGGAGCGCACCATCTTCTTCAAGGACGACGGCAACTACAAGACCCGCGCCGA
GGTGAAGTTCGAGGGCGACACCCTGGTGAACCGCATCGAGCTGAAGGGCATCGACTTCAAGG
AGGACGGCAACATCCTGGGGCACAAGCTGGAGTACAACTACAACAGCCACAACGTCTATATC
ATGGCCGACAAGCAGAAGAACGGCATCAAGGTGAACTTCAAGATCCGCCACAACATCGAGGA
CGGCAGCGTGCAGCTCGCCGACCACTACCAGCAGAACACCCCCATCGGCGACGGCCCCGTGC
TGCTGCCCGACAACCACTACCTGAGCACCCAGTCCGCCCTGAGCAAAGACCCCAACGAGAAG
CGCGATCACATGGTCCTGCTGGAGTTCGTGACCGCCGCCGGGATCACTCTCGGCATGGACGA
GCTGTACAAGTGAAGATCCAGTACTGCAGAATTCAGCTCAGGTAGATAGTCTCATTAGAGAA
CCACGTAATTGGGTCAACCCGTTAATACCGGTCCACCAAGCCCTGGAAATCTCTAGTAATCT
ACATGCACAAAACCGCTGTTATTCTAAATACGCGATGTTAAATATTATTAGTCTCATTAACA
AACCACGCAACTATGTCAACCCGCTAATACCGGTCTAATAGGCCCTGGAAATTGTTAATAAA
TAATATTGTAACATCAAACCATATATATGTAATAGTCTCATCACTAACCCACGCAACTGTGT
CAATCCGTTAATACCGGTCCAACAGGCCCTGGAAATTAGTGGCAATAACATATATATGTCAA
CCCTGCTCATTGGTTTAATTGAGCGCATTTAGTGTTGTGTGATCTTGCGCGATAAATGCTGA
CGTGAAAACGTTGCGTATTGCTACAACACTTGGTTAGCTATTTAGCTTTACTAATCAAGACG
CCGTCGTGCAGCCCACAAAAGTCTAGATACGTCACAGGAGAGCATACGCTAGGTCGCGTTGA
CTATCCTTATATATGACCTGCAATGGTGAGCGAGCTGATTAAGGAGAACATGCACATGAAGC
TGTACATGGAGGGCACCGTGAACAACCACCACTTCAAGTGCACATCCGAGGGCGAAGGCAAG
CCCTACGAGGGCACCCAGACCATGAGAATCAAGGCGGTCGAGGGCGGCCCTCTCCCCTTCGC
CTTCGACATCCTGGCTACCAGCTTCATGTACGGCAGCAAAACCTTCATCAACCACACCCAGG
GCATCCCCGACTTCTTTAAGCAGTCCTTCCCCGAGGGCTTCACATGGGAGAGAGTCACCACA
TACGAAGACGGGGGCGTGCTGACCGCTACCCAGGACACCAGCCTCCAGGACGGCTGCCTCAT
CTACAACGTCAAGATCAGAGGGGTGAACTTCCCATCCAACGGCCCTGTGATGCAGAAGAAAA
CACTCGGCTGGGAGGCCTCCACCGAGACCCTGTACCCCGCTGACGGCGGCCTGGAAGGCAGA
GCCGACATGGCCCTGAAGCTCGTGGGCGGGGGCCACCTGATCTGCAACTTGAAGACCACATA
CAGATCCAAGAAACCCGCTAAGAACCTCAAGATGCCCGGCGTCTACTATGTGGACAGAAGAC
TGGAAAGAATCAAGGAGGCCGACAAAGAGACCTACGTCGAGCAGCACGAGGTGGCTGTGGCC
AGATACTGCGACCTCCCTAGCAAACTGGGGCACAGATAAGCGGCCGCGAATTAATTCGCCTT
AGACATGACTGTTCCTCAGTTCAAGTTGGGCACTTACGAGAAGACCGGTCTTGCTAGATTCT
AATCAAGAGGATGTCAGAATGCCATTTGCCTGAGAGATGCAGGCTTCATTTTTGATACTTTT
TTATTTGTAACCTATATAGTATAGGATTTTTTTTGTCATTTTGTTTCTTCTCGTACGAGCTT
GCTC

Sequence of the expression cassette of p9KAox-GTK (1966 bp)
CAGAAGGAAGCTGCCCTGTCTTAAACCTTTTTTTTTATCATCATTATTAGCTTACTTTCATAATTGCGACTGGTT
CCAATTGACAAGCTTTTGATTTTAACGACTTTTAACGACAACTTGAGAAGATCAAAAAACAACTAATTATTCGAA

GGATCCAAACGATGGTGAGCAAGGGCGAGGAGCTGTTCACCGGGGTGGTGCCCATCCTGGTCGAGCTGGACGGCG
ACGTAAACGGCCACAAGTTCAGCGTGTCCGGCGAGGGCGAGGGCGATGCCACCTACGGCAAGCTGACCCTGAAGT
TCATCTGCACCACCGGCAAGCTGCCCGTGCCCTGGCCCACCCTCGTGACCACCCTGACCTACGGCGTGCAGTGCT
TCAGCCGCTACCCCGACCACATGAAGCAGCACGACTTCTTCAAGTCCGCCATGCCCGAAGGCTACGTCCAGGAGC
GCACCATCTTCTTCAAGGACGACGGCAACTACAAGACCCGCGCCGAGGTGAAGTTCGAGGGCGACACCCTGGTGA
ACCGCATCGAGCTGAAGGGCATCGACTTCAAGGAGGACGGCAACATCCTGGGGCACAAGCTGGAGTACAACTACA
ACAGCCACAACGTCTATATCATGGCCGACAAGCAGAAGAACGGCATCAAGGTGAACTTCAAGATCCGCCACAACA
TCGAGGACGGCAGCGTGCAGCTCGCCGACCACTACCAGCAGAACACCCCCATCGGCGACGGCCCCGTGCTGCTGC
CCGACAACCACTACCTGAGCACCCAGTCCGCCCTGAGCAAAGACCCCAACGAGAAGCGCGATCACATGGTCCTGC
TGGAGTTCGTGACCGCCGCCGGGATCACTCTCGGCATGGACGAGCTGTACAAGTGAAGATCCAGTACTGCAGAAT
TCAGCTCAGGTAAATAACAAATCTCAACACAACATATACAAAACAAACGAATCTCAAGCAATCAAGCATTCTACT
TCTATTGCAGCAATTTAAATCATTTCTTTTAAAGCAAAAGCAATTTTCTGAAAATTTTCACCATTTACGAACGAT
AGCAATGGTGAGCGAGCTGATTAAGGAGAACATGCACATGAAGCTGTACATGGAGGGCACCGTGAACAACCACCA
CTTCAAGTGCACATCCGAGGGCGAAGGCAAGCCCTACGAGGGCACCCAGACCATGAGAATCAAGGCGGTCGAGGG
CGGCCCTCTCCCCTTCGCCTTCGACATCCTGGCTACCAGCTTCATGTACGGCAGCAAAACCTTCATCAACCACAC
CCAGGGCATCCCCGACTTCTTTAAGCAGTCCTTCCCCGAGGGCTTCACATGGGAGAGAGTCACCACATACGAAGA
CGGGGGCGTGCTGACCGCTACCCAGGACACCAGCCTCCAGGACGGCTGCCTCATCTACAACGTCAAGATCAGAGG
GGTGAACTTCCCATCCAACGGCCCTGTGATGCAGAAGAAAACACTCGGCTGGGAGGCCTCCACCGAGACCCTGTA
CCCCGCTGACGGCGGCCTGGAAGGCAGAGCCGACATGGCCCTGAAGCTCGTGGGCGGGGGCCACCTGATCTGCAA
CTTGAAGACCACATACAGATCCAAGAAACCCGCTAAGAACCTCAAGATGCCCGGCGTCTACTATGTGGACAGAAG
ACTGGAAAGAATCAAGGAGGCCGACAAAGAGACCTACGTCGAGCAGCACGAGGTGGCTGTGGCCAGATACTGCGA
CCTCCCTAGCAAACTGGGGCACAGATAAGCGGCCGCGAATTAATTCGCCTTAGACATGACTGTTCCTCAGTTCAA
GTTGGGCACTTACGAGAAGACCGGTCTTGCTAGATTCTAATCAAGAGGATGTCAGAATGCCATTTGCCTGAGAGA
TGCAGGCTTCATTTTTGATACTTTTTTATTTGTAACCTATATAGTATAGGATTTTTTTTGTCATTTTGTTTCTTC
TCGTACGAGCTTGCTC

Sequence of the expression cassette of p9KAox-GEK (2409 bp)

CAGAAGGAAGCTGCCCTGTCTTAAACCTTTTTTTTTATCATCATTATTAGCTTACTTTCATAATTGCGACTGGTT
CCAATTGACAAGCTTTTGATTTTAACGACTTTTAACGACAACTTGAGAAGATCAAAAAACAACTAATTATTCGAA
GGATCCAAACGATGGTGAGCAAGGGCGAGGAGCTGTTCACCGGGGTGGTGCCCATCCTGGTCGAGCTGGACGGCG
ACGTAAACGGCCACAAGTTCAGCGTGTCCGGCGAGGGCGAGGGCGATGCCACCTACGGCAAGCTGACCCTGAAGT
TCATCTGCACCACCGGCAAGCTGCCCGTGCCCTGGCCCACCCTCGTGACCACCCTGACCTACGGCGTGCAGTGCT
TCAGCCGCTACCCCGACCACATGAAGCAGCACGACTTCTTCAAGTCCGCCATGCCCGAAGGCTACGTCCAGGAGC
GCACCATCTTCTTCAAGGACGACGGCAACTACAAGACCCGCGCCGAGGTGAAGTTCGAGGGCGACACCCTGGTGA
ACCGCATCGAGCTGAAGGGCATCGACTTCAAGGAGGACGGCAACATCCTGGGGCACAAGCTGGAGTACAACTACA
ACAGCCACAACGTCTATATCATGGCCGACAAGCAGAAGAACGGCATCAAGGTGAACTTCAAGATCCGCCACAACA
TCGAGGACGGCAGCGTGCAGCTCGCCGACCACTACCAGCAGAACACCCCCATCGGCGACGGCCCCGTGCTGCTGC
CCGACAACCACTACCTGAGCACCCAGTCCGCCCTGAGCAAAGACCCCAACGAGAAGCGCGATCACATGGTCCTGC
TGGAGTTCGTGACCGCCGCCGGGATCACTCTCGGCATGGACGAGCTGTACAAGTGAAGATCCAGTACTGCAGAAT
TCAGCTCAGGTGGCAAATTCCGCCCCTCTCCCTCCCCCCCCCCTAACGTTACTGGCCGAAGCCGCTTGGAATAAG
GCCGGTGTGCGTTTGTCTATATGTTATTTTCCACCATATTGCCGTCTTTTGGCAATGTGAGGGCCCGGAAACCTG
GCCCTGTCTTCTTGACGAGCATTCCTAGGGGTCTTTCCCCTCTCGCCAAAGGAATGCAAGGTCTGTTGAATGTCG
TGAAGGAAGCAGTTCCTCTGGAAGCTTCTTGAAGACAAACAACGTCTGTAGCGACCCTTTGCAGGCAGCGGAACC
CCCCACCTGGCGACAGGTGCCTCTGCGGCCAAAAGCCACGTGTATAAGATACACCTGCAAAGGCGGCACAACCCC
AGTGCCACGTTGTGAGTTGGATAGTTGTGGAAAGAGTCAAATGGCTCTCCTCAAGCGTATTCAACAAGGGGCTGA
AGGATGCCCAGAAGGTACCCCATTGTATGGGATCTGATCTGGGGCCTCGGTGCACATGCTTTACATGTGTTTAGT
CGAGGTTAAAAAACGTCTAGGCCCCCCGAACCACGGGGACGTGGTTTTCCTTTGAAAAACACGATAATACCATG
GTGAGCGAGCTGATTAAGGAGAACATGCACATGAAGCTGTACATGGAGGGCACCGTGAACAACCACCACTTCAAG
TGCACATCCGAGGGCGAAGGCAAGCCCTACGAGGGCACCCAGACCATGAGAATCAAGGCGGTCGAGGGCGGCCCT
CTCCCCTTCGCCTTCGACATCCTGGCTACCAGCTTCATGTACGGCAGCAAAACCTTCATCAACCACACCCAGGGC
ATCCCCGACTTCTTTAAGCAGTCCTTCCCCGAGGGCTTCACATGGGAGAGAGTCACCACATACGAAGACGGGGGC
GTGCTGACCGCTACCCAGGACACCAGCCTCCAGGACGGCTGCCTCATCTACAACGTCAAGATCAGAGGGGTGAAC
TTCCCATCCAACGGCCCTGTGATGCAGAAGAAAACACTCGGCTGGGAGGCCTCCACCGAGACCCTGTACCCCGCT
GACGGCGGCCTGGAAGGCAGAGCCGACATGGCCCTGAAGCTCGTGGGCGGGGGCCACCTGATCTGCAACTTGAAG
ACCACATACAGATCCAAGAAACCCGCTAAGAACCTCAAGATGCCCGGCGTCTACTATGTGGACAGAAGACTGGAA
AGAATCAAGGAGGCCGACAAAGAGACCTACGTCGAGCAGCACGAGGTGGCTGTGGCCAGATACTGCGACCTCCCT
AGCAAACTGGGGCACAGATAAGCGGCCGCGAATTAATTCGCCTTAGACATGACTGTTCCTCAGTTCAAGTTGGGC
ACTTACGAGAAGACCGGTCTTGCTAGATTCTAATCAAGAGGATGTCAGAATGCCATTTGCCTGAGAGATGCAGGC
TTCATTTTTGATACTTTTTTATTTGTAACCTATATAGTATAGGATTTTTTTTGTCATTTTGTTTCTTCTCGTACG
AGCTTGCTC

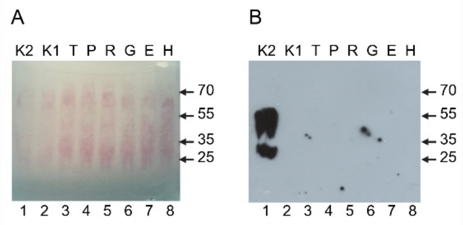

Figure S3. Immunoblot analysis of mKate2 protein production in *K. phaffii* cells carrying mono- and bicistronic expression cassettes under the control of the P_{AOX1} promoter. **(A)** Ponceau S staining of the membrane after protein transfer (Materials and Methods). **(B)** The same immunoblot as in panel A of the main figure (Figure 3), at a longer exposure. Lysates of induced cells were probed with anti-mKate2 antibodies (Materials and Methods). Lanes: K2, monocistronic mKate2; K1, EGFP-expression control (with a frameshift in the mKate2 ORF); T, P, R, G, E, H, bicistronic constructs carrying the TEV, PVY, RhPV, GPR1, EMCV, and HCV IRES elements, respectively. Arrows indicate the positions of molecular weight markers in kDa.

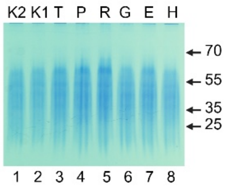

Figure S4. Total-protein loading control for lysates of *K. phaffii* cells carrying mono- and bicistronic expression cassettes under the control of the P_{AOX1} promoter. Coomassie G-250 staining of a duplicate non-denaturing gel, used as a loading control for the samples analyzed by in-gel EGFP fluorescence. Lanes: K2, monocistronic mKate2; K1, EGFP-expression control (with a frameshift in the mKate2 ORF); T, P, R, G, E, H, bicistronic constructs carrying the TEV, PVY, RhPV, GPR1, EMCV, and HCV IRES elements, respectively. Arrows indicate the positions of molecular weight markers in kDa.
